# Genetic basis of enhanced stress resistance in long‐lived mutants highlights key role of innate immunity in determining longevity

**DOI:** 10.1111/acel.13740

**Published:** 2022-12-13

**Authors:** Sonja K. Soo, Annika Traa, Zenith D. Rudich, Alibek Moldakozhayev, Meeta Mistry, Jeremy M. Van Raamsdonk

**Affiliations:** ^1^ Department of Neurology and Neurosurgery McGill University Montreal Quebec Canada; ^2^ Metabolic Disorders and Complications Program, and Brain Repair and Integrative Neuroscience Program Research Institute of the McGill University Health Centre Montreal Quebec Canada; ^3^ Bioinformatics Core, Harvard School of Public Health Harvard Medical School Boston Massachusetts USA; ^4^ Division of Experimental Medicine, Department of Medicine McGill University Montreal Quebec Canada

**Keywords:** aging, *C. elegans*, DAF‐16/FOXO, genetics, innate immunity, lifespan, SKN‐1/NRF2, stress resistance

## Abstract

Mutations that extend lifespan are associated with enhanced resistance to stress. To better understand the molecular mechanisms underlying this relationship, we directly compared lifespan extension, resistance to external stressors, and gene expression in a panel of nine long‐lived *Caenorhabditis elegans* mutants from different pathways of lifespan extension. All of the examined long‐lived mutants exhibited increased resistance to one or more types of stress. Resistance to each of the examined types of stress had a significant, positive correlation with lifespan, with bacterial pathogen resistance showing the strongest relationship. Analysis of transcriptional changes indicated that all of the examined long‐lived mutants showed a significant upregulation of multiple stress response pathways. Interestingly, there was a very significant overlap between genes highly correlated with stress resistance and genes highly correlated with longevity, suggesting that the same genetic pathways drive both phenotypes. This was especially true for genes correlated with bacterial pathogen resistance, which showed an 84% overlap with genes correlated with lifespan. To further explore the relationship between innate immunity and longevity, we disrupted the p38‐mediated innate immune signaling pathway in each of the long‐lived mutants and found that this pathway is required for lifespan extension in eight of nine mutants. Overall, our results demonstrate a strong correlation between stress resistance and longevity that results from the high degree of overlap in genes contributing to each phenotype. Moreover, these findings demonstrate the importance of the innate immune system in lifespan determination and indicate that the same underlying genes drive both immunity and longevity.

## Graphical abstract

To advance our understanding of the relationship between stress resistance and lifespan, we measured lifespan, stress resistance, and gene expression in a panel of nine long‐lived mutants in *Caenorhabditis elegans*. All of the long‐lived mutants exhibit enhanced resistance to at least one external stressor resulting from significant upregulation of multiple stress response pathways. This work indicates that the same genetic pathways contribute to stress resistance and lifespan and demonstrates the importance of innate immune signaling and other stress response pathways in determining longevity.

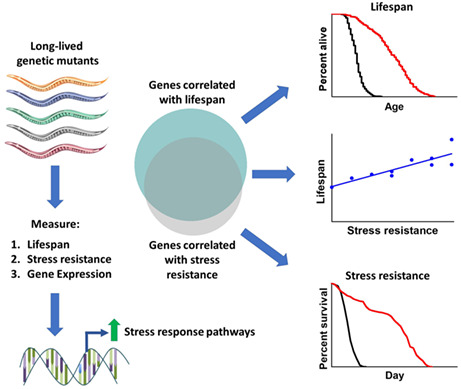

**Abbreviations**
ANOVA: analysis of variance
Cyto‐UPR: cytoplasmic unfolded protein response
ER‐UPR: endoplasmic reticulum unfolded protein response
FDR: false discovery rate
FUdR: fluorodeoxyuridine
GO: gene ontology
mitoUPR: mitochondrial unfolded protein response
NGM: nematode growth medium
PCA: principal component analysis
RNAi: RNA interference
RNA‐seq: RNA sequencing
ROS: reactive oxygen species
rRNA: ribosomal RNA
SEM: standard error of the mean
UV: ultraviolent

## 1 INTRODUCTION

Paradigm‐shifting work in the worm *Caenorhabditis elegans* has identified single‐gene mutations that significantly extend lifespan, thereby demonstrating a clear contribution of genetics to lifespan determination. Since the first genes to increase lifespan were identified in *C. elegans* (Friedman & Johnson, 1988; Kenyon et al., 1993; Klass, 1977; Wong et al., 1995), single‐gene mutations have also been shown to extend lifespan in other model organisms including yeast, flies, and mice (Clancy et al., 2001; Fabrizio et al., 2001; Holzenberger et al., 2003). Importantly, many of the genes or interventions that extend lifespan are evolutionarily conserved (Kenyon, 2005).

The availability of genetic mutants with extended lifespan has facilitated investigation into the mechanisms underlying their increased longevity, and the categorization of genetic mutants into specific pathways of lifespan extension. These pathways include decreased insulin/IGF‐1 signaling (Friedman & Johnson, 1988; Kenyon et al., 1993), mild impairment of mitochondrial function (Feng et al., 2001; Lakowski & Hekimi, 1996; Yang & Hekimi, 2010), dietary restriction (Lakowski & Hekimi, 1998), germ line inhibition (Hsin & Kenyon, 1999), reduced chemosensation (Apfeld & Kenyon, 1999), decreased translation (Hansen et al., 2007; Syntichaki et al., 2007), and increased mitochondrial reactive oxygen species (ROS) (Van Raamsdonk & Hekimi, 2009).

In exploring factors contributing to longevity, it has been observed that many long‐lived mutants exhibit increased resistance to at least one type of external stressor. The best characterized example is the long‐lived insulin/IGF‐1 receptor mutant *daf‐2*, which has increased resistance to oxidative stress, heat stress, osmotic stress, anoxia, heavy metals, and bacterial pathogens (Barsyte et al., 2001; Dues et al., 2019; Garsin et al., 2003; Honda & Honda, 1999; Lithgow et al., 1995). However, many counterexamples also exist. Mutations in the mitochondrial superoxide dismutase gene (*sod‐2*) increase lifespan but decrease resistance to oxidative stress (Van Raamsdonk & Hekimi, 2009). The inverse also occurs where mutations that increase stress resistance result in decreased lifespan, such as the constitutive activation of the mitochondrial unfolded protein response (mitoUPR) transcription factor ATFS‐1 (Bennett et al., 2014; Pellegrino et al., 2014; Soo et al., 2021). Decreasing stress resistance in *daf‐2* mutants through disruption of *gpdh‐1/2* or *nhl‐1* diminishes stress resistance but increases lifespan (Dues et al., 2019). Thus, although stress resistance and lifespan are positively correlated, there are examples of genes that affect these phenotypes in opposite directions or that affect one but not the other.

A relationship between stress resistance and aging is also supported by the observation that resistance to multiple external stressors declines with age (Dues et al., 2016), at least in part due to a genetically programmed downregulation of stress response pathways (Labbadia & Morimoto, 2015; Van Raamsdonk, 2017). Importantly, a positive relationship between stress resistance and lifespan is conserved across species (Arking et al., 1991; Johnson et al., 2001; Salmon et al., 2005) and resistance to physiological stress is proposed to be one of the eight hallmarks of aging (López‐otín et al., 2013).

In this study, we measured stress resistance in nine long‐lived mutants from multiple different pathways of lifespan extension. We then compared the stress resistance of each mutant to their lifespan extension and changes in gene expression across the genome. By quantifying all of these factors within the same study, we were able to directly compare lifespan and stress resistance across all of the long‐lived mutants and examine the genetic underpinnings of stress resistance in these long‐lived strains. We found that all nine of the long‐lived mutants that we examined have increased resistance to at least one external stressor and that all six of the examined types of stress resistance are significantly correlated with lifespan. In exploring the underlying mechanisms, we found that all of the long‐lived mutants exhibit upregulation of genetic targets of multiple stress response pathways. Finally, we found that genes correlated with stress resistance exhibit a highly significant overlap with genes correlated with lifespan. Overall, this work advances our understanding of the relationship between stress resistance and longevity and demonstrates that the genetic pathways that contribute to these two phenotypes are highly overlapping, especially in the case of innate immune signaling.

## 2 RESULTS

### 2.1 Long‐lived mutants show different magnitudes of lifespan extension

To determine the extent to which different types of stress resistance are correlated with longevity and which types of stress resistance exhibit the highest correlation, we quantified resistance to stress and lifespan in nine long‐lived mutants representing different pathways of lifespan extension. Measuring the stress resistance of these mutants together in the same assay allowed us to compare the relative magnitude of stress resistance with the lifespan extension of each mutant.

The mutants that we examined included: *daf‐2* worms, which have decreased insulin/IGF‐1 signaling (Kenyon et al., 1993); *eat‐2* worms, which are a model of dietary restriction (Lakowski & Hekimi, 1998); *ife‐2* worms, which have decreased translation (Hansen et al., 2007; Syntichaki et al., 2007); *clk‐1*, *isp‐1* and *nuo‐6* worms, which have mild impairment of mitochondrial function (Feng et al., 2001; Lakowski & Hekimi, 1996; Wong et al., 1995; Yang & Hekimi, 2010); *sod‐2* worms, which have increased mitochondrial ROS (Van Raamsdonk & Hekimi, 2009); *osm‐5* worms, which have reduced chemosensation (Apfeld & Kenyon, 1999); and *glp‐1* worms, which are a model of germ line ablation (Hsin & Kenyon, 1999). Since *glp‐1* worms need to be grown at 25°C during development for the temperature‐sensitive mutation to induce sterility and extend lifespan, a separate wild‐type control grown at 25°C during development was used in all experiments.

**FIGURE 1:**
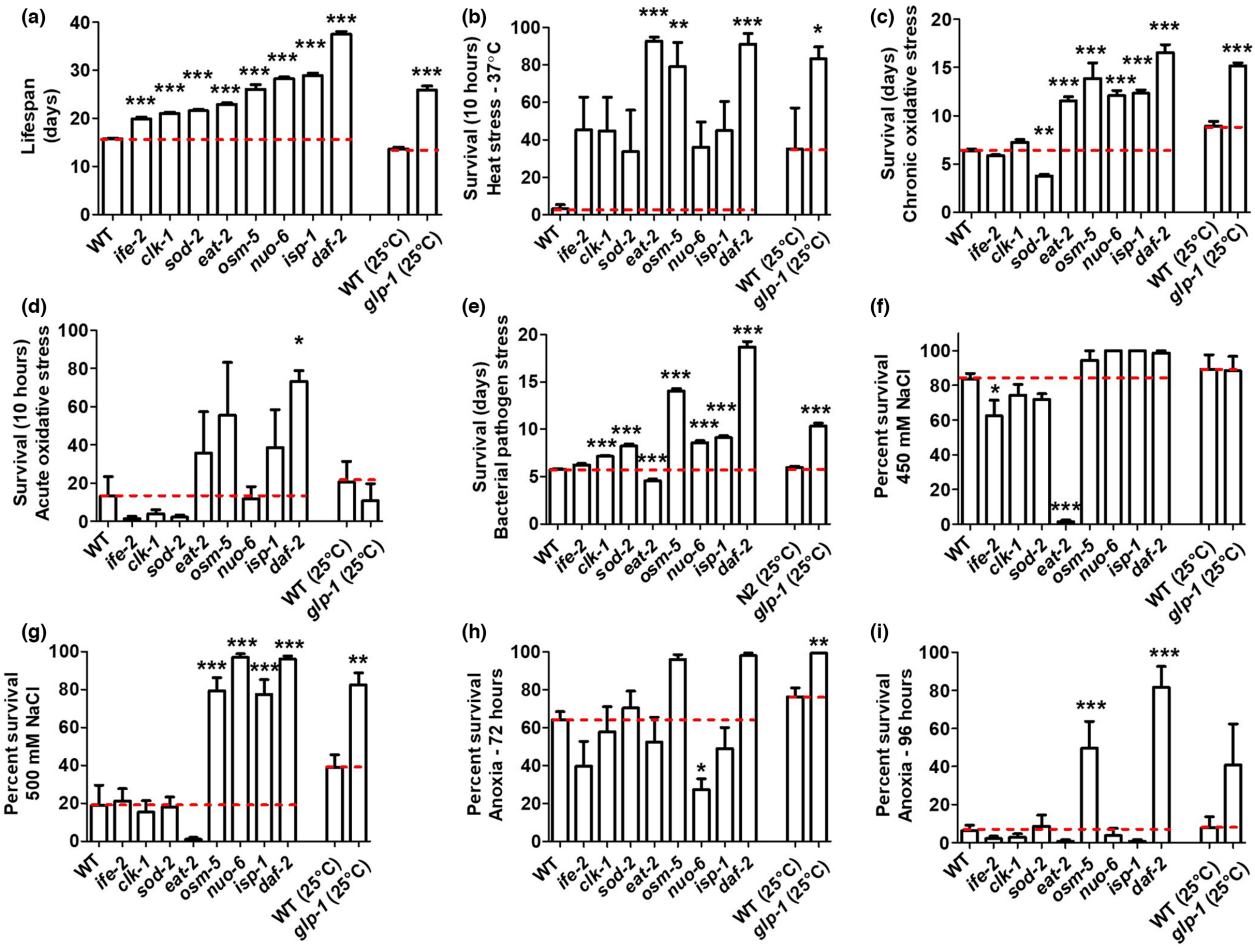
Long‐lived mutants have increased resistance to multiple external stressors. (a) The lifespans of nine different long‐lived mutants were compared directly in the same assay. There was a range in the magnitude of lifespan extension observed across the nine mutants (for complete lifespan plots, see Figure S1). (b) All of the long‐lived mutants exhibit increased resistance to heat stress at 37°C (for full time course, see Figure S2). (c) Six of the nine long‐lived mutants have increased resistance to chronic oxidative stress (4 mM paraquat), while *sod‐2* mutants have decreased resistance (for full time course, see Figure S3). (d) Four of the long‐lived mutants show increased resistance to acute oxidative stress (300 μM juglone), while four mutants have decreased resistance (for full time course, see Figure S4). (e) All of the long‐lived mutants except for *eat‐2* mutants exhibit increased resistance to bacterial pathogen stress (for full time course, see Figure S5). Resistance to osmotic stress was determined by exposing worms to 450 or 500 mM NaCl for 48 h. (f) At 450 mM NaCl, *ife‐2* and *eat‐2* mutants show less resistance to osmotic stress compared with wild‐type worms. (g) At 500 mM NaCl, *osm‐5*, *nuo‐6*, *isp‐1*, *daf‐2*, and *glp‐1* show increased resistance to osmotic stress. Resistance to anoxia was measured after 72 and 96 h of anoxia. (h) After 72 h of anoxia, *nuo‐6* mutants show decreased resistance to anoxia compared with wild‐type worms, while *glp‐1* shows increased resistance compared wild‐type controls. (i) After 96 h of anoxia, *osm‐5* and *daf‐2* worms show increased resistance to anoxia compared with wild‐type worms. For *glp‐1* worms and their wild‐type controls, worms were grown at 25°C during development and were shifted to 20°C at adulthood. Error bars represent SEM. *p < 0.05, **p < 0.01, ***p < 0.001. A minimum of three biological replicates were performed. Statistical analysis was performed using a one‐way ANOVA with Dunnett's multiple comparison test, except for *glp‐1* which was compared with its wild‐type control using a Student's *t*‐test. The data in this figure are a summary of the results from the full longitudinal survival assays that are presented in Figures S1–S5. Because this figure does not contain all of the time points present in the supplemental figures, some strains that have significantly increased resistance to stress when all of the time points are considered (Figures S1–S5) do not show a significant increase in this figure.

We confirmed that all of the long‐lived mutant strains have increased lifespan (Figure 1a; Figure S1). Importantly, by simultaneously measuring the lifespans of these strains in the same assay, the magnitude of lifespan extension can be directly compared between the long‐lived mutants. In order of smallest to largest degree of lifespan extension were *ife‐2* worms (26.3%), *clk‐1* worms (33.4%), *sod‐2* worms (37.2%), *eat‐2* worms (45.6%), *osm‐5* worms (65.4%), *nuo‐6* worms (79.2%), *isp‐1* (83.8%), *glp‐1* worms (89.2%), and *daf‐2* worms (138.4%). For the remaining figures, these strains will be arranged in order of the magnitude of lifespan extension, to easily visualize the extent to which stress resistance and expression of stress response genes correlates with lifespan.

### 2.2 All long‐lived mutants have increased resistance to one or more external stressor

To identify which types of stress resistance are most strongly correlated with longevity, we next quantified the relative resistance of the nine long‐lived mutant strains to six external stressors using well‐established stress paradigms including heat stress (37°C), chronic oxidative stress (4 mM paraquat), acute oxidative stress (300 μM juglone), bacterial pathogens (*P. aeruginosa* strain PA14), osmotic stress (450 or 500 mM NaCl), and anoxia (72 or 96 h). Comparing the stress resistance of these mutants in the same assay also enabled us to identify genetic pathways driving the increased stress resistance in these long‐lived mutants.

We found that all of the long‐lived mutants showed increased resistance to heat stress compared with the wild‐type worms, with *eat‐2*, *osm‐5*, *daf‐2*, and *glp‐1* being the most resistant (Figure S2; 10‐h time point is shown in Figure 1b). The majority of the long‐lived mutants also exhibited increased resistance to chronic oxidative stress, except for *ife‐2* and *sod‐2* worms, which have significantly decreased resistance (Figure S3; average survival is shown in Figure 1c). By contrast, only *eat‐2*, *osm‐5*, *isp‐1*, and *daf‐2* worms have increased resistance to acute oxidative stress, while *ife‐2*, *clk‐1*, *sod‐2*, and *glp‐1* worms are more sensitive (Figure S4; 10‐h time point is shown in Figure 1d). After exposure to bacterial pathogens, all of the long‐lived mutants except for *eat‐2* worms showed increased survival, with *osm‐5* and *daf‐2* worms having the greatest survival (Figure S5; average survival is shown in Figure 1e). The increased sensitivity of *eat‐2* worms to bacterial pathogen stress, which others have reported previously (Kumar et al., 2019), likely results at least partially from the fact that they accumulate live bacteria at a greater rate than wild‐type worms, which may be due to a deficit in grinding bacteria in their pharynx (Kumar et al., 2019).

In the osmotic stress assay, *ife‐2* and *eat‐2* show decreased resistance (Figure 1f), while *osm‐5*, *nuo‐6*, *isp‐1*, *daf‐2*, and *glp‐1* have increased resistance (Figure 1g). Finally, under conditions of anoxia, *nuo‐6* worms have decreased survival while *glp‐1*, *osm‐5*, and *daf‐2* worms have increased survival compared with wild‐type worms (Figure 1h,i).

Overall, all of the long‐lived mutants are resistant to at least one type of stressor (Table S1). Some of the long‐lived mutants (*ife‐2*, *sod‐2*, *eat‐2*, and *nuo‐6*) also show decreased resistance to certain external stressors. *daf‐2* mutants are resistant to all six stressors tested and, in most assays, exhibited the greatest survival.

### 2.3 Resistance to multiple different external stressors is significantly correlated with lifespan

**FIGURE 2:**
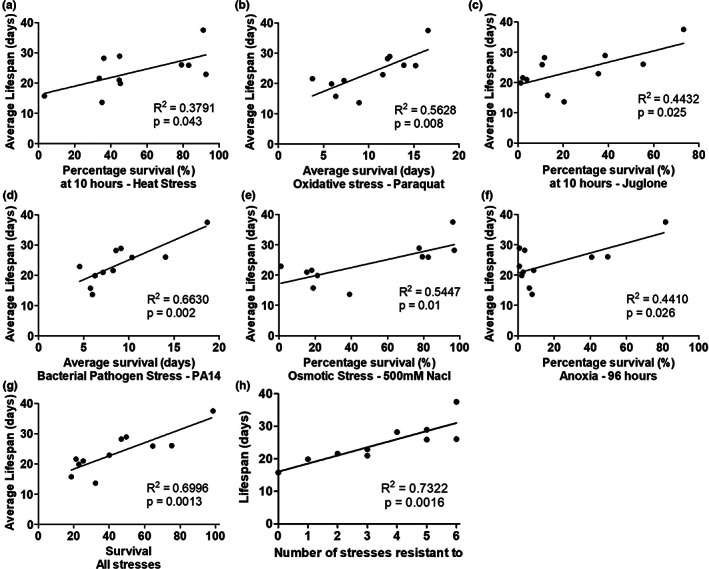
Resistance to multiple types of stress is significantly correlated with longevity. To determine the extent to which each type of stress resistance is correlated with lifespan, we compared the magnitude of lifespan extension to the percentage or duration of survival following exposure to exogenous stressors. There is a significant correlation with each of the six different types of stress resistance and lifespan including heat stress (a), chronic oxidative stress (b), acute oxidative stress (c), bacterial pathogens (d), osmotic stress (e), and anoxia (f). The highest degree of correlation was observed for bacterial pathogen stress. Combining all six types of stress resistance together into a combined survival score only slightly increased the degree of correlation with lifespan (g). The highest correlation with lifespan was achieved by using the number of stresses that a particular mutant is resistant to (h). Note that in panels b and d average survival was graphed as these stress assays were monitored until completion, while the other panels examine a specific time point. *p < .05, **p < .01, ***p < .001.

To determine which types of stress resistance are correlated with lifespan, we compared the survival after exposure to each of the examined stressors to the average lifespan of the mutant. All six stressors had a significant, positive correlation between survival under stress and longevity with *R* ^2^ values ranging from 0.3791 to 0.6630 (Figure 2a–f). These results are consistent with a previous study, which found a positive correlation between heat stress resistance and lifespan (*R* ^2^ = 0.36), and UV resistance and lifespan (*R* ^2^ = 0.42) (Johnson et al., 2001). Resistance to bacterial pathogens (Figure 2d, *R* ^2^ = 0.6330) and resistance to chronic oxidative damage (Figure 2b, *R* ^2^ = 0.5628) exhibited the greatest correlation with lifespan, which may at least partially be due to the chronic nature of these assays.

To evaluate whether resistance to multiple types of stressors is more predictive of lifespan than resistance to individual stressors, we combined the stress resistance data using two different approaches. First, we summed the relative stress resistance scores by setting the maximum stress resistance for each assay to 100% and expressed the stress resistance of each strain as a percentage of this maximum. This allowed us to combine the results from the six stress resistance assays at equal weight to generate an overall stress survival score. This combined stress survival score had only a marginally higher *R* ^2^ value than the highest single stress *R* ^2^ value (Figure 2g, *R* ^2^ = 0.6996). Second, we counted the numbers of stressors for which a strain showed significantly increased survival. Combining the stress resistance data in this way resulted in a slightly higher *R* ^2^ value of 0.7322 (Figure 2h). Overall, these results indicate that stress resistance is positively correlated with lifespan.

### 2.4 Long‐lived mutants exhibit upregulation of genetic targets of multiple stress response pathways

**FIGURE 3:**
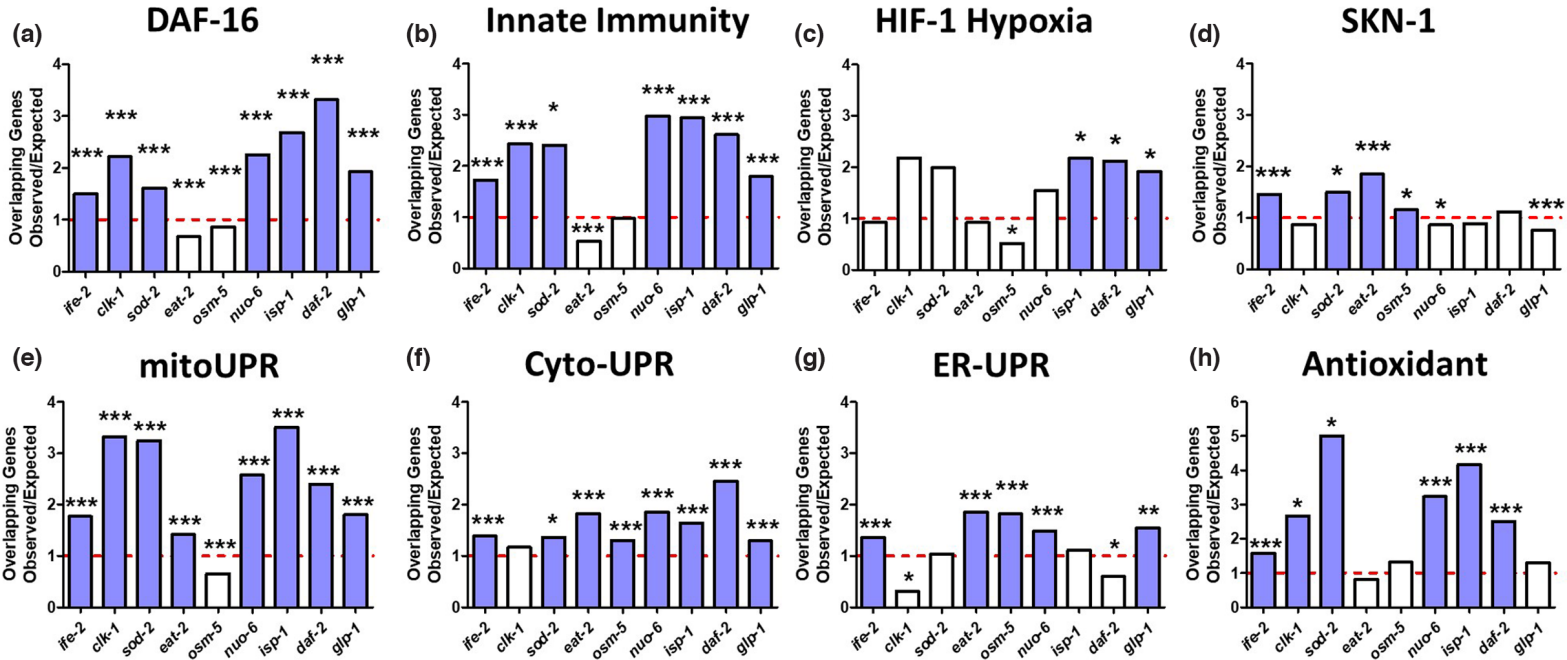
Long‐lived mutants exhibit upregulation of genetic targets of multiple stress response pathways. Gene expression in the long‐lived mutant strains was examined by RNA sequencing (RNA‐seq) of six biological replicate per genotype of prefertile day 1 young adult worms. Differentially expressed genes that are significantly upregulated in the long‐lived mutant strains were compared with genes that are upregulated by the activation of different stress response pathways including the DAF‐16‐mediated stress response (a), the p38‐regulated innate immune signaling pathway (b), the HIF‐1‐mediated hypoxia response (c), the SKN‐1‐mediated stress response (d), the mitochondrial unfolded protein response (mitoUPR) (e), the cytoplasmic unfolded protein response (Cyto‐UPR) (f), the ER‐mediated unfolded protein response (ER‐UPR) (g), and antioxidant gene expression (h). For each mutant and stress pathway, we calculated the number of genes expected in the overlap between the two gene sets if all of the genes in each gene set were randomly selected. We then divided the number of overlapping genes that we actually observed by the number of overlapping genes expected by chance to generate the observed/expected ratio, which is illustrated in each graph. An observed/expected ratio of greater than 1 indicates that genes upregulated in a particular mutant are enriched in genes that are part of the particular stress response pathway being examined. Each of the eight stress response pathways (panels a–h) is upregulated in multiple long‐lived mutants. All of the long‐lived mutants show a significant enrichment of genes involved in multiple stress response pathways. Blue bars indicate a statistically significant enrichments of stress response genes. *p < .05, **p < 0.01, ***p < 0.001. *p*‐Value represents the significance of overlap between genes upregulated in the indicated long‐lived mutant and genes upregulated by the activation of the indicated stress response pathway.

Having shown that all nine long‐lived mutants that we tested display increased resistance to stress, we sought to explore the underlying mechanisms leading to stress resistance. We hypothesized that these mutants upregulate one or more stress response pathways. To evaluate the activation of each stress response pathway, we compared differentially expressed genes in the long‐lived mutant strains to the target genes of established stress response pathways including the DAF‐16‐mediated stress response pathway (Figure 3a; Tepper et al., 2013), the p38‐mediated innate immunity pathway (Figure 3b; Fletcher et al., 2019), the HIF‐1‐mediated hypoxia response pathway (Figure 3c; Shen, Nettleton, et al., 2005), the SKN‐1‐mediated oxidative stress response pathway (Figure 3d; Steinbaugh et al., 2015), the mitochondrial unfolded protein response (mitoUPR) pathway (Figure 3e; Ewald et al., 2015; Nargund et al., 2012), the cytoplasmic unfolded protein response (Cyto‐UPR) pathway (Figure 3f; Li et al., 2016; Sural et al., 2019), the ER‐mediated unfolded protein response (ER‐UPR) pathway (Figure 3g; Shen, Ellis, et al., 2005), and antioxidant gene expression (Figure 3h; Soo et al., 2021). Lists of the target genes from each stress response pathway and the associated references are provided in Table S2.

In comparing the differentially expressed genes in each long‐lived mutant to genes that are significantly modulated by activation by a specific stress response pathway, we determined the number of overlapping genes and then divided this number by the predicted number of overlapping genes expected if the genes were chosen at random to produce a ratio of observed/expected. An observed/expected ratio of greater than 1 indicates that genes upregulated in a particular mutant are enriched in genes that are part of the particular stress response pathway being examined (this can be thought of as fold enrichment). All of the long‐lived mutants had a significant enrichment of genetic targets from three or more stress response pathways (Figure 3). In particular, we found that *ife‐2* showed significant enrichment of genes from 7 stress response pathways; *sod‐2*, *nuo‐6*, *isp‐1*, *daf‐2*, and *glp‐1* showed enrichment of genes from 6 pathways; *clk‐1* and *eat‐2* showed enrichment of genes from 4 pathways; and *osm‐5* showed enrichment of genes from 3 pathways. Interestingly, the most strongly enriched stress response pathway differed between strains (Figure S6).

Among the stress response pathways, targets of the mitoUPR and the Cyto‐UPR were enriched in eight long‐lived mutants (Figure 3e,f). Targets of the DAF‐16‐mediated stress pathway and the p38‐mediated innate immunity pathway were enriched in seven mutants (Figure 3a,b). Antioxidant genes were enriched in six mutants (Figure 3h). Targets of the ER‐UPR were enriched in five mutants (Figure 3g). Targets of the SKN‐1‐mediated oxidative stress response were enriched in four mutants (Figure 3d). Genes from the HIF‐1‐mediated hypoxia response were enriched in three mutants (Figure 3c).

Finally, we examined the extent to which the ratio of observed/expected overlapping genes with each stress response pathway is correlated with longevity. Ratios for overlapping genes for both the DAF‐16‐mediated stress response pathway and the Cyto‐UPR pathway have a significant correlation with lifespan (Figure S7). These results suggest that the increased stress resistance in the long‐lived mutants is caused by the activation of multiple stress response pathways under unstressed conditions.

### 2.5 Genetic correlates of resistance to stress

To determine which individual genes are contributing to each type of stress resistance, we analyzed RNA‐seq data from each long‐lived mutant to identify genes whose expression is correlated with resistance to a specific stressor. Resistance to each external stressor had a strong correlation to the expression of specific genes, with the maximum *R* ^2^ values ranging from 0.71 for heat stress resistance to 0.91 for bacterial pathogen resistance. This suggests that genetic factors are contributing to stress resistance.

We found that at least 71 genes (heat stress) and as many as 1115 genes (bacterial pathogens) exhibited a significant, positive correlation with each type of stress resistance, while 67 genes (heat stress) to 1014 genes (osmotic stress) exhibit a significant negative correlation with the different types of stress resistance (Figure S8, Table S3). There were far more genes positively correlated with bacterial pathogen resistance than negatively correlated (7.4‐fold more), similar to what we observed for lifespan. By contrast, resistance to osmotic stress had many more negatively correlated genes than positively correlated genes (2.2‐fold more).

To determine the extent to which the same genes are driving different types of stress resistance, we determined the overlap between genes correlated with one type of stress resistance with genes correlated with each other type of stress resistance. We compared genes that are both positively (Figure S9a) and negatively (Figure S9b) correlated with each type of stress resistance with the goal of determining the extent to which the genes correlated with stress resistance would be correlated with multiple types of stress resistance or be specific to certain types of stress resistance. We also wanted to determine which types of stress resistance show the greatest overlap of correlated genes. We found that there are 20 genes correlated with resistance to five different stressors along with 69, 270, 666, and 2759 genes correlated with four, three, two, and one stressor, respectively (Table S4). For positively correlated genes, the greatest degree of overlap occurred between genes correlated with resistance to bacterial pathogens and genes correlated with resistance to anoxia (412 genes, 63% overlap) and between genes correlated with resistance to bacterial pathogens and genes correlated with resistance to acute oxidative stress (343 genes, 62% overlap) (Figure S9a). It is important to note that this result is complicated by the fact that each gene set has different numbers of genes ranging from 71 to 1115, and thus, it is important to consider both the number of overlapping genes and the percentage of overlap.

For negatively correlated genes, the greatest overlap occurred between genes correlated with resistance to acute and chronic oxidative stress (100 genes, 36% overlap) and between genes correlated with resistance to osmotic stress resistance and genes correlated with bacterial pathogen resistance (73 genes, 49% overlap) (Figure S9b). Combined, this suggests that similar genetic pathways can contribute to multiple types of stress resistance. In addition, there also exists some overlap in genes that are upregulated by the activation of each stress response pathway (Figure S10; Soo et al., 2021).

To understand the mechanisms by which genes correlated with stress resistance are acting to enhance resistance to stress, we combined all of the genes that exhibited a significant positive correlation to one or more types of stress and looked for overrepresentation of these genes within Gene Ontology (GO) terms according to biological process, molecular function, or cellular component (Figure S11). There was an approximately threefold enrichment within the biological processes “mitochondrial electron transport,” “aerobic respiration,” and “regulation of response to oxidative stress” (Figure S11a). For molecular functions, there was a 1.5–threefold enrichment of “chitin binding,” structural constituent of cuticle,” “structural constituent of ribosome” and “oxidoreductase activity” (Figure S11b). This suggests that enhancing the worm's physical barrier to the environment promotes resistance to stress. Finally, for cellular components, there was a 1.5–threefold enrichment of “mitochondrial respiratory chain complex I,” “cytosolic ribosomal subunit,” “collagen trimer,” and “extracellular region” (Figure S11c), again suggesting the importance of the mitochondria and physical barrier in enhancing resistance to external stressors.

### 2.6 Individual genes correlated with stress resistance affect the survival of exogenous stressors

**FIGURE 4:**
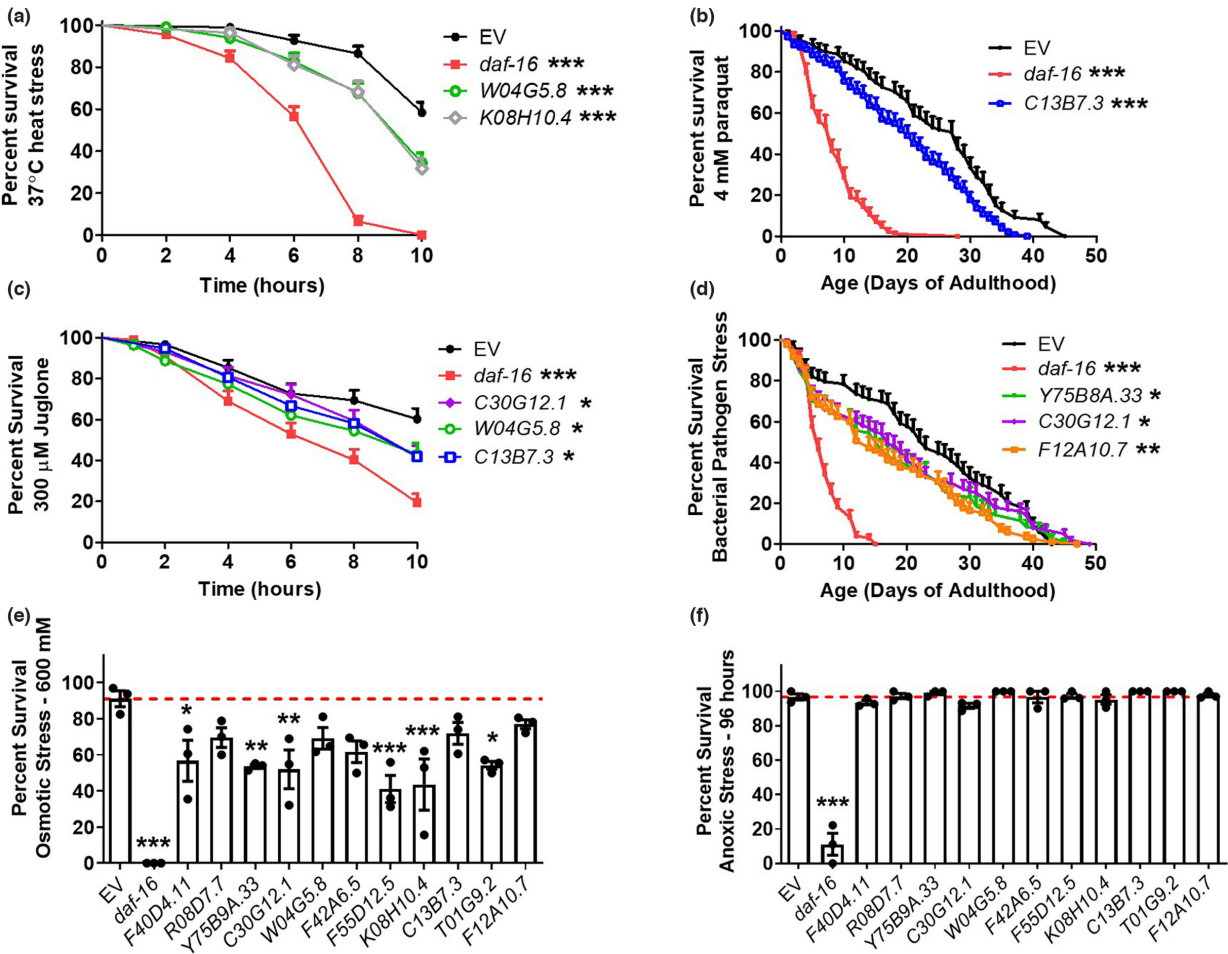
Knockdown of genes correlated with resistance to stress decreases resistance to exogenous stressors. Eleven genes that exhibited a significant positive correlation with resistance to exogenous stressors were knocked down in stress‐resistant, long‐lived *daf‐2* mutant worms using RNAi before examining resistance to heat stress (a; 37°C), chronic oxidative stress (b; 4 mM paraquat), acute oxidative stress (c; 300 μM juglone), bacterial pathogens (d; *P. aeruginosa* strain PA14), osmotic stress (e; 600 mM), or anoxia (f; 96 h). *daf‐16* RNAi was used as a positive control. With the exception of anoxic stress, knockdown of one or more of the genes correlated with stress resistance resulted in decreased survival of exogenous stressors. This indicates that genes correlated with stress resistance can impact resistance to stress individually. Three biological replicates were performed. Statistical analysis was performed using the Gehan–Greslow–Wilcoxon test in panels a–d and a one‐way ANOVA with Dunnett's multiple comparison test in panels e and f. Error bars represent SEM. *p < 0.05, **p < 0.01, ***p < 0.001. For panels, a–d only RNAi clones that significantly decreased stress resistance are shown so that graphs are not overcrowded. Results for all 11 genes are provided in Table S5.

Having identified numerous genes that are correlated with stress resistance, we next sought to determine whether disruption of these genes individually can have an impact on resistance to stress. For this purpose, we used RNA interference (RNAi) to knockdown a set of 11 genes that were either correlated with all types of stress resistance (*F40D4.11*, *R08D7.7*, *Y75B8A.33*, and *C30G12.1*) or specific types of stress resistance (*W04G5.8*, *F42A6.5*, *F55D12.5*, *K08H10.4*, *C13B7.3*, *T01G9.2*, and *F12A10.7*). We performed these experiments in stress‐resistant *daf‐2* worms so that there would be a larger window to observe a detrimental effect on stress resistance and used *daf‐16* RNAi as a control, which we have previously shown to decrease stress resistance in *daf‐2* mutants (Dues et al., 2019). We found that knockdown of nine of the 11 stress‐correlated genes examined resulted in a significant decrease in resistance to one or more exogenous stressor (Figure 4, Table S5). However, it is also important to note that knockdown of two of the 11 genes did not significantly affect resistance to any of the stresses that we tested and that the genes did not necessarily have a significant effect on all of the types of stress resistance that their expression was correlated with. This may be the result of many correlated genes having effects that are too small to demonstrate a statistically significant effect, or it is possible that these genes are acting as markers of stress resistance through co‐regulation or other mechanisms but do not actually contribute to stress resistance. Nonetheless, our results clearly indicate that at least some of the genes that we identified as being correlated with stress resistance can impact resistance to stress individually. Further studies will be required to see which of the other correlated genes are affecting stress resistance and to elucidate the mechanisms by which each gene enhances resistance to stress.

### 2.7 Genes contributing to stress resistance exhibit significant enrichment of genes contributing to longevity

**FIGURE 5:**
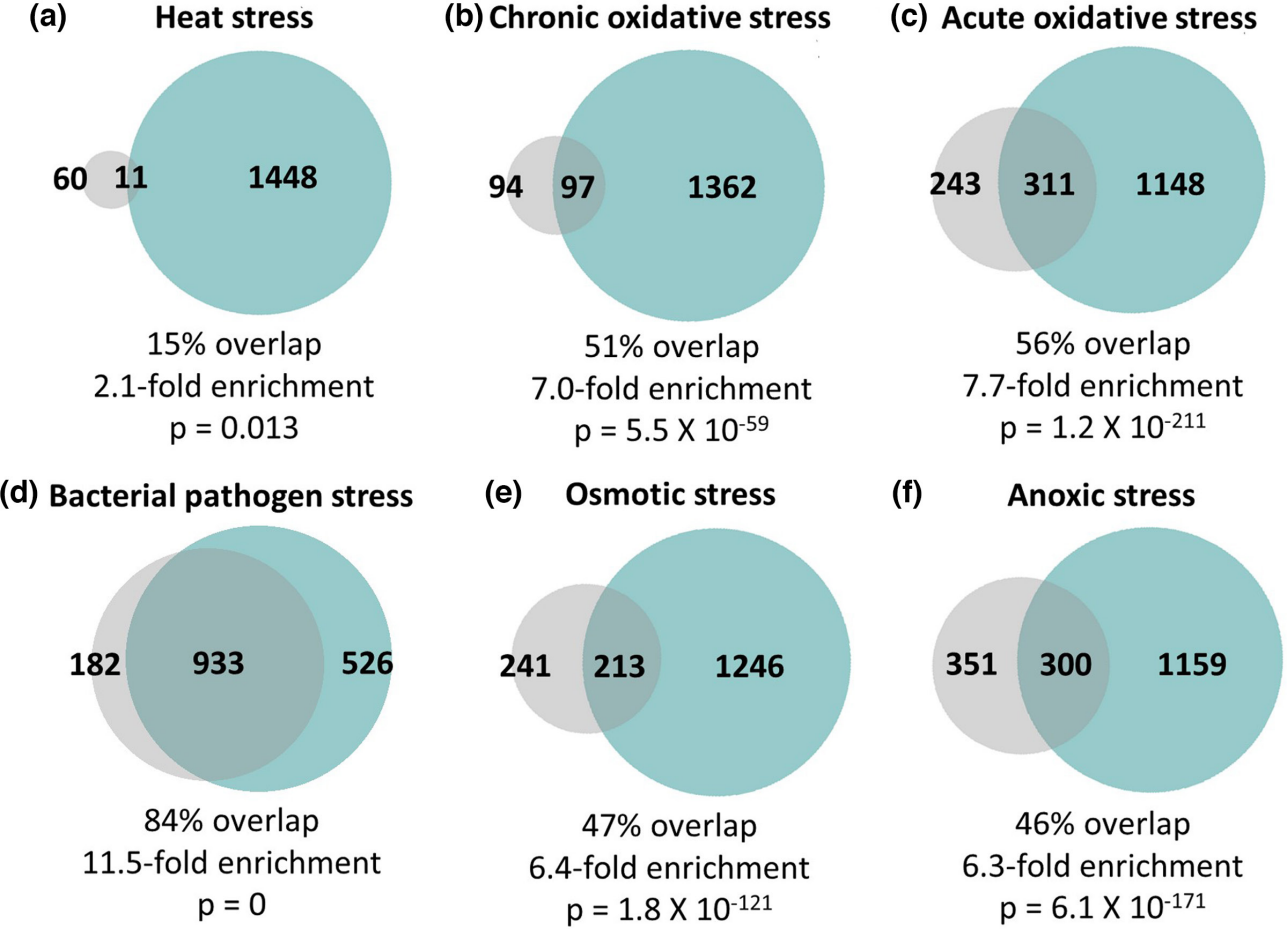
Highly significant overlap between genes that are correlated with resistance to stress and genes that are correlated with lifespan extension. In examining the degree of overlap between genes that are significantly correlated with lifespan (teal circle) and genes that are significantly correlated with resistance to stress (gray circle), we observed a significant degree of overlap with each of the six types of stress resistance that we examined including heat stress (a; 37°C), chronic oxidative stress (b; 4 mM paraquat), acute oxidative stress (c; 300 μM juglone), bacterial pathogen (d; *P. aeruginosa*), osmotic stress (e; 450–500 mM NaCl), and anoxic stress (f; 72–96 h). The degree of enrichment ranged from 2.1 fold up to 11.5 fold with percent overlap between 15% and 84%. The highest degree of overlap with genes correlated with lifespan was with genes correlated with bacterial pathogen stress survival. The total number of genes that are unique to each circle (gene set) and number of overlapping genes are indicated. Percent overlap was calculated by dividing the number of overlapping genes by the number of genes positively correlated with each type of stress resistance. Enrichment was calculated as the number of overlapping genes observed divided by the number of overlapping genes expected by picking genes at random. The *p*‐value indicates the significance of the difference between observed and expected numbers of overlapping genes.

To determine the extent to which the same genetic pathways are driving stress resistance and longevity on a broad scale, we compared genes that are correlated with each type of stress resistance to genes that are correlated with lifespan. We found that there was a significant degree of overlap between genes positively correlated with stress resistance and genes positively correlated with longevity ranging from 15% overlap (2.1‐fold enrichment) to 84% overlap (11.5‐fold enrichment), with the greatest degree of overlap occurring for resistance to bacterial pathogens (Figure 5; Figure S12). Genes that are negatively correlated with stress resistance were also negatively correlated with lifespan, though to a lesser extent than positively correlated genes (Figure S13). These findings suggest that the same genes and genetic pathways are contributing to both stress resistance and lifespan.

### 2.8 Innate immune signaling pathway is required for longevity of long‐lived mutants

**FIGURE 6:**
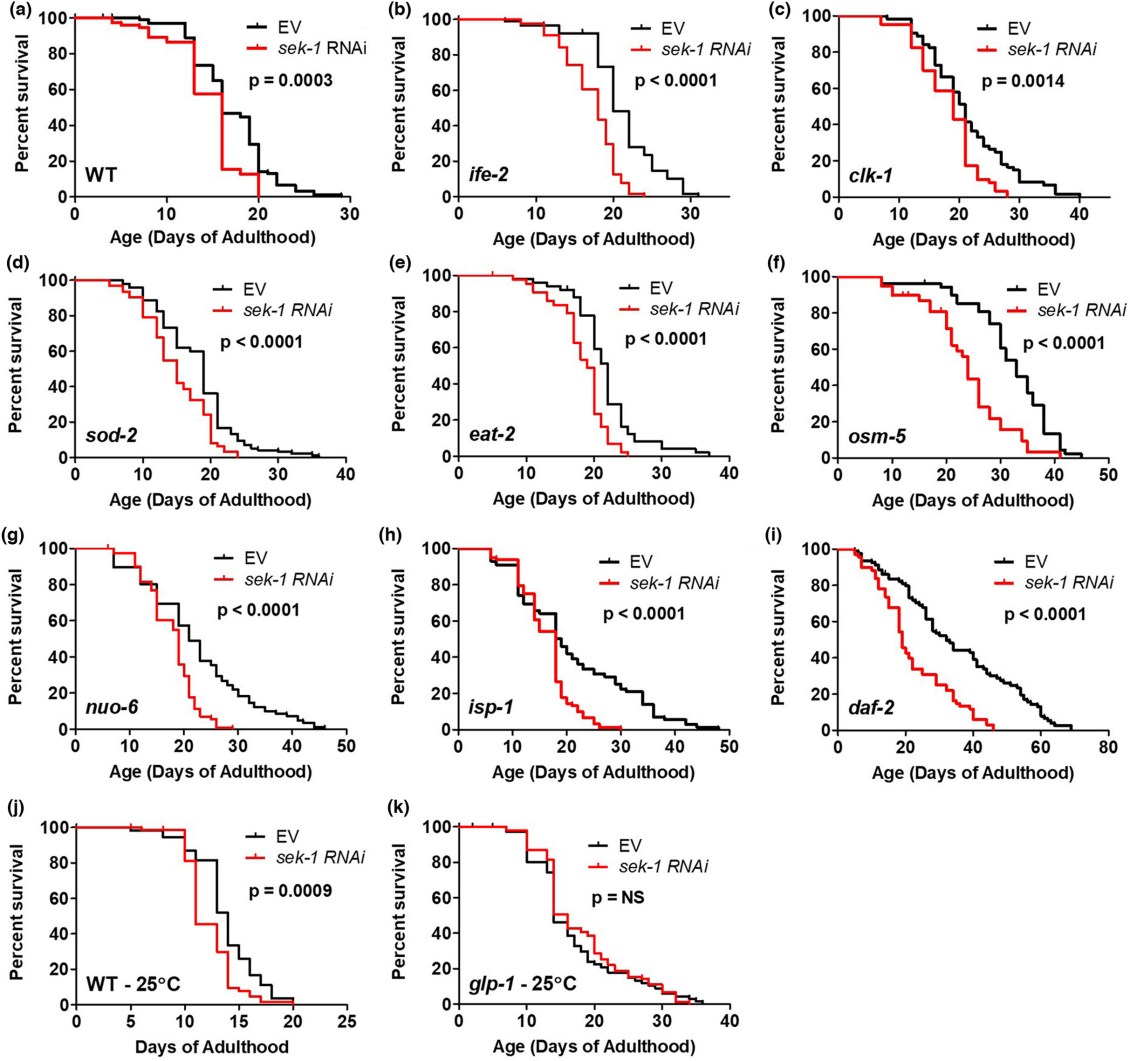
p38‐mediated innate immune signaling pathway is required for lifespan extension in long‐lived genetic mutants. Innate immune signaling was disrupted by knocking down the expression of *sek‐1* using RNAi in a panel of nine long‐lived genetic mutants. Knocking down expression of *sek‐1* significantly decreased the lifespan of WT worms (a), *ife‐2* mutants (b), *clk‐1* mutants (c), *sod‐2* mutants (d), *eat‐2* mutants (e), *osm‐5* mutants (f), *nuo‐6* mutants (g), *isp‐1* mutants (h), and *daf‐2* mutants (i). While wild‐type worms grown at 25°C during development showed decreased lifespan when *sek‐1* was disrupted (j), *glp‐1* lifespan was not affected by *sek‐1* RNAi (k). These results indicate that the p38‐mediated innate immune signaling pathway is required for lifespan extension. Three biological replicates were performed. Statistical significance was assessed using the log‐rank test.

Of all of the types of stress resistance we examined, we observed the greatest impact of bacterial pathogen resistance on lifespan, including the strongest correlation (Figure 2) and the greatest overlap with genes correlated with lifespan (Figure 5). We have recently shown that the p38‐mediated innate immune signaling pathway is required for lifespan extension resulting from mild impairment of mitochondrial function (Campos et al., 2021), while others have shown that this pathway is needed for the long lifespan of *daf‐2* mutants and worms treated with dietary restriction (Wu et al., 2019). Accordingly, we sought to determine the extent to which this pathway is required for lifespan extension in each of the nine long‐lived mutants that we examined. To do this, we disrupted the p38‐mediated innate immune signaling pathway by knocking down *sek‐1* using RNAi. We found that *sek‐1* RNAi decreased the lifespan of eight of the nine long‐lived mutants that we examined and also decreased the lifespan of wild‐type worms (Figure 6). While this result is consistent with a role for innate immune signaling in the lifespan extension of these mutants, this conclusion is complicated by the fact that *sek‐1* RNAi also decreased the lifespan of wild‐type worms (see discussion).

## 3 DISCUSSION

The survival of an organism depends upon its ability to resist physiological stress. Animals not only have to survive exogenous stressors in their environment such as changes in temperature, changes in oxygen availability, and presence of pathogens, but also internal stressors produced as by‐products of cellular processes. The activation of stress pathways in response to physiological stressors is important to maintain cellular homeostasis amidst fluctuations in the environment and is evolutionarily conserved. By quantifying resistance to multiple external stressors in long‐lived *C. elegans* mutants and analyzing their gene expression, we found that long‐lived mutants have increased resistance to stress due to upregulation of multiple stress response pathways. Genes correlated with stress resistance exhibit a highly significant overlap with genes correlated with lifespan, suggesting that similar genetic pathways contribute to both phenotypes. Moreover, our results indicate that genetic pathways governing resistance to bacterial pathogens are the most important for promoting longevity.

### 3.1 Increased resistance to stress is associated with extended longevity

Since the discovery of genetic mutant strains that have extended lifespan, multiple groups have examined their ability to withstand different external stressors (Zhou et al., 2011). *daf‐2* mutants are the most well characterized in this regard and have increased resistance to oxidative stress, heat stress, osmotic stress, anoxia, heavy metals, and bacterial pathogens (Barsyte et al., 2001; Dues et al., 2019; Garsin et al., 2003; Honda & Honda, 1999; Lithgow et al., 1995). Similarly, *eat‐2* mutants have increased resistance to heat stress (Hansen et al., 2007); *ife‐2* mutants have increased resistance to heat stress and oxidative stress (Hansen et al., 2007; Syntichaki et al., 2007); *glp‐1* mutants have increased resistance to bacterial pathogens, heat stress, and oxidative stress (Amrit et al., 2019; TeKippe & Aballay, 2010); *clk‐1* mutants have increased resistance to chronic oxidative stress (Schaar et al., 2015); *isp‐1* mutants have increased resistance to heat stress, oxidative stress, osmotic stress, and bacterial pathogens (Campos et al., 2021; Dues et al., 2017); and *nuo‐6* worms have increased resistance to heat stress, oxidative stress, osmotic stress, and bacterial pathogens (Campos et al., 2021; Wu et al., 2018).

In this study, we extended previous findings by comprehensively examining resistance to six of the most well‐studied stress paradigms in nine long‐lived mutants from multiple different pathways of lifespan extension (Figure 1). Our results show that all of the examined long‐lived mutants have increased resistance to at least one external stressor, with some mutants having increased resistance to all examined stressors (*daf‐2* and, *osm‐5*), while other mutants are resistant to only one (*ife‐2*). Importantly, our study permitted a direct comparison of the magnitude of stress resistance among this panel of long‐lived mutants and its relationship with the magnitude of lifespan extension and underlying changes in gene expression. The number of stressors that a mutant is resistant to is correlated with the magnitude of their lifespan extension. While this result is consistent with the conclusion that resistance to a wide array of stressors is beneficial for longevity, the lifespan of these worms is extended under laboratory conditions, which are believed to be relatively unstressful. Thus, an alternative explanation for the correlation between stress resistance and lifespan is that the same genetic pathways contribute to both aging and stress resistance, which is supported by our gene expression studies.

All of the types of stress resistance that we examined showed a significant positive correlation with lifespan, with resistance to bacterial pathogens having the strongest relationship with lifespan (Figure 2). This extends the findings of a previous study that showed a positive correlation between lifespan and thermotolerance, UV resistance, and juglone resistance using different sets of mutants (Johnson et al., 2001). In our study, all of the long‐lived mutants showed increased resistance to heat stress, suggesting that this type of stress resistance may be more important for longevity than other types. However, others have observed decreased resistance to heat stress in long‐lived *daf‐28* mutants (Johnson et al., 2001; Malone et al., 1996). Thus, for each of the external stressors we examined, there are one or more long‐lived mutants that exhibit decreased survival. This indicates that increasing lifespan is not sufficient to provide protection against any specific external stressor.

Interestingly, we did not always observe the same outcome for acute and chronic oxidative stress assays (Table S1; e.g., *clk‐1*, *glp‐1*). Our previous work indicates that this difference is not accounted for by the fact that different compounds were used to induce oxidative stress (paraquat for chronic versus juglone for acute), since *clk‐1* worms showed increased sensitivity to acute oxidative stress resulting from exposure to high doses of paraquat or juglone (Schaar et al., 2015). One possibility is that surviving the higher dose of oxidative stress used in the acute oxidative stress assay is dependent on having upregulation of stress response pathways prior to stress exposure. On the contrary, the lower dose of oxidative stress used in the chronic oxidative stress assay may be dependent on both the baseline level of the activation of stress response pathways and the ability of the mutant to upregulate stress response pathways in response to stress. Another possibility is that the level of stress response pathway upregulation is important. Low‐level activation may be sufficient to protect against chronic lower‐dose oxidative stress, while higher‐level activation may be needed to protect against acute higher‐dose oxidative stress.

### 3.2 Stress resistance can be experimentally dissociated from lifespan

To untangle the relationship between stress resistance and longevity, researchers have experimentally manipulated stress resistance pathways and examined the resulting effects on lifespan. While disrupting stress response pathways typically decreases lifespan in both wild‐type worms and long‐lived mutants, there are multiple exceptions.

Mutations can decrease resistance to one or multiple stressors, but have no effect on lifespan, or even increase lifespan. Disruption of the mitoUPR transcription factor ATFS‐1 decreases resistance to multiple stressors (Wu et al., 2018), but is reported to either have no effect on lifespan (Lan et al., 2019; Wu et al., 2018) or increase lifespan (Bennett et al., 2014). Deletion of *sod‐2* decreases resistance to oxidative stress, but increases lifespan (Van Raamsdonk & Hekimi, 2009). Similarly, disruption of *clk‐1* decreases resistance to acute oxidative stress but increases lifespan (Schaar et al., 2015). Here, we show that *ife‐2* mutants have decreased resistance to oxidative stress and osmotic stress, *eat‐2* mutants have decreased resistance to osmotic stress and bacterial pathogens, and *glp‐1* worms have decreased resistance to acute oxidative stress, but all three mutants still show increased lifespan (Table S1). Worms with mutations disrupting all five superoxide dismutase genes (*sod*) have decreased resistance to multiple stressors, but do not have decreased lifespan (Dues et al., 2019; Van Raamsdonk & Hekimi, 2012). Disruption of both glycerol‐3‐phosphate dehydrogenase (*gpdh*) genes or deletion of the putative E3 ubiquitin ligase gene *nhl‐1* makes *daf‐2* worms more sensitive to osmotic stress, but increases their lifespan (Dues et al., 2019). Disruption of the p38‐mediated innate immunity pathway through deletion of *pmk‐1* decreases resistance to bacterial pathogens but does not affect lifespan (Dues et al., 2019). Combined, these examples demonstrate that decreasing resistance to stress is not sufficient to decrease lifespan.

There are also examples in which increasing stress resistance does not increase lifespan. Mutations in *daf‐4* or *daf‐7* result in increased resistance to heat stress, but do not extend longevity (Lithgow et al., 1995). Constitutive activation of ATFS‐1 increases resistance to multiple stressors but decreases lifespan (Soo et al., 2021). Disruption of the transcription elongation regulator TCER‐1 increases resistance to bacterial pathogens, heat stress, and oxidative stress in *glp‐1* mutants but decreases their lifespan (Amrit et al., 2019; Ghazi et al., 2009). Thus, increasing stress resistance is not sufficient to increase lifespan. Combined, these examples indicate that stress resistance can be experimentally dissociated from longevity.

### 3.3 Long‐lived mutants exhibit upregulation of multiple stress response pathways

To better understand the molecular mechanisms underlying the increased stress resistance in the long‐lived mutants, we compared gene expression in these mutants to genes modulated by the activation of established stress response pathways. We found that all nine of the long‐lived mutants exhibit a statistically significant enrichment of genes that are upregulated by the activation of multiple stress response pathways. The greatest number of mutants showed enrichment of genetic targets of the mitoUPR pathway (8 of 9), the cyto‐UPR pathway (8 of 9), the DAF‐16‐mediated stress response pathway (7 of 9), and the p38‐mediated innate immunity pathway (7 of 9), suggesting that these stress response pathways may be more important contributors to stress resistance and longevity of these mutants. These findings extend our previous work in which we observed a significant upregulation of DAF‐16 target genes (Senchuk et al., 2018), mitoUPR target genes (Wu et al., 2018), innate immunity genes (Campos et al., 2021), and antioxidant genes (Dues et al., 2017; Schaar et al., 2015) in the long‐lived mitochondrial mutants *clk‐1*, *isp‐1*, and *nuo‐6*. In addition to examining the number of genes that are significantly modulated in each stress response pathway, it is also important to consider other factors including the magnitude of upregulation or downregulation of each stress response gene, which specific stress response genes are differentially expressed, and the period of time when the genes are being differentially expressed.

Although the mitoUPR pathway is activated in the greatest number of long‐lived mutants, this does not necessarily indicate that mitoUPR activation is sufficient for longevity. In a study that performed a genome‐wide RNAi screen to identify RNAi clones that activate the mitoUPR, it was found that these clones could increase or decrease lifespan (Bennett et al., 2014). Similarly, we and others have shown that constitutive activation of the mitoUPR transcription factor ATFS‐1 shortens lifespan (Bennett et al., 2014; Soo et al., 2021), which could be due to diverting energy or important cellular machinery away from other pathways that contribute to longevity. It is likely that the level of mitoUPR activation as well as other factors determine whether or not mitoUPR activation increases lifespan.

### 3.4 The same genetic pathways affect stress resistance and longevity

From our results here and previous studies, the significant correlation between stress resistance and lifespan cannot be explained by models in which increased stress resistance causes extended longevity or vice versa, as multiple counterexamples exist. To gain insight into this relationship, we examined the extent to which the genes driving stress resistance overlap with genes driving longevity. For each of the six types of stress resistance examined, there was a statistically significant overlap between genes correlated with stress resistance and genes correlated with longevity, suggesting that many of the same genes that contribute to stress resistance also contribute to longevity. Based on the high degree of overlap between genes controlling stress resistance and genes controlling longevity, we propose that the significant correlation between stress resistance and aging is due to a large group of genes that contribute to both stress resistance and longevity (Figure S14). Whenever one of these overlapping genes is modulated, both phenotypes are affected. There are also sets of genes that contribute only to lifespan or only to stress resistance. Modulation of these genes can affect lifespan independently of stress resistance, or vice versa, thereby allowing for the experimental dissociation of stress resistance and lifespan.

### 3.5 Innate immunity and longevity

Aging is associated with the dysregulation of the immune system (Shaw et al., 2013). In *C. elegans*, aging results in increased susceptibility to bacterial infections as well as a decline in the activation of the p38‐mediated innate immunity pathway (Dues et al., 2016; Youngman et al., 2011), which is the main pathway for pathogen defense (Pukkila‐Worley & Ausubel, 2012). Of all of the types of stress resistance that we examined, we observed the strongest relationship between resistance to bacterial pathogens and longevity. Lifespan was most highly correlated with resistance to bacterial pathogens (Figure 2), and genes correlated with lifespan had the highest overlap with genes correlated with resistance to bacterial pathogens (Figure 5; 84%). In addition, seven of the nine long‐lived mutants we examined exhibited a significant upregulation of target genes of the p38‐mediated innate immune signaling pathway (Figure 3). Combined, these results indicate that the activation of pathways enabling the survival of bacterial pathogen exposure promotes longevity.

The importance of innate immunity for longevity is also supported by our finding the disruption of p38‐mediated innate immune signaling through RNAi against *sek‐1* decreases the lifespan of almost all of the long‐lived mutants examined (Figure 6). Our observation that *sek‐1* RNAi also decreased the lifespan of wild‐type worms raises the possibility that disruption of innate immune signaling is having a nonspecific detrimental effect on lifespan. In this case, additional information is helpful in defining the contribution of innate immune signaling to lifespan extension. We and others have shown that mutations in *sek‐1*, which do not affect wild‐type lifespan, decrease the extended lifespans of *nuo‐6*, *isp‐1*, *daf‐2*, and *eat‐2* mutants (Campos et al., 2021; Wu et al., 2019). We have also shown that other proteins in the innate immune signaling pathway (NSY‐1, PMK‐1, and ATF‐7) are required for the long lifespan of *nuo‐6* and *isp‐1* mutants (Campos et al., 2021). We and others have shown that PMK‐1 and ATF‐7 are required for *daf‐2* longevity (Dues et al., 2019; Troemel et al., 2006; Wu et al., 2019; Zarse et al., 2012). Similarly, we have observed that PMK‐1 is required for the long lifespan of *clk‐1* and *sod‐2* mutants (Figure S15). Combined, these results indicate that innate immune signaling is important for lifespan extension in many of the long‐lived mutants studied.

It is important to note that the *sek‐1* RNAi experiments were conducted under unstressful laboratory conditions in the absence of bacterial pathogens, suggesting that the beneficial effect of the p38‐mediated innate immune signaling pathway on longevity is not necessarily related to its ability to protect against bacterial pathogens. In support of this conclusion, we and others have shown that the p38‐mediated innate immune signaling pathway is still required for the extended lifespan of long‐lived mutants when they are being fed nonproliferating bacteria (Campos et al., 2021; Wu et al., 2019). In future, it would be interesting to examine the effects of the innate immune signaling pathway on the lifespan of these long‐lived mutants using killed bacteria and a genetic mutation to disrupt innate immune signaling.

## 4 CONCLUSION

Different pathways of lifespan extension all induce enhanced resistance to at least one external stressor and significant upregulation of multiple stress response pathways in long‐lived *C. elegans* mutants. Longevity is significantly and positively correlated with all six types of stress resistance examined and shows the strongest correlation with resistance to bacterial pathogens. By identifying the genes that are most highly correlated with each type of stress resistance, we found that the same genetic pathways contribute to both resistance to stress and longevity and that the strongest relationship exists between resistance to bacterial pathogens and lifespan. This indicates that the correlation between stress resistance and lifespan is due to a large group of genes that contribute to both phenotypes. Overall, this work demonstrates a role for stress response pathways in determining lifespan and emphasizes the importance of innate immune signaling for longevity.

## 5 EXPERIMENTAL PROCEDURES

### 5.1 Strains and maintenance

All *C. elegans* strains were obtained from the Caenorhabditis Genetics Center (CGC): N2 (wild‐type), *ife‐2 (ok306)*, *clk‐1(qm30)*, *sod‐2(ok1030)*, *eat‐2(ad1116)*, *osm‐5(p813)*, *nuo‐6(qm200)*, *isp‐1(qm150)*, *daf‐2(e1370)*, and *glp‐1(e2141)*. All strains were grown and maintained in nematode‐grown medium (NGM) plates at 20°C except *glp‐1(e2141)*, which was maintained at 20°C, but grown at 25°C to induce sterility and lifespan extension in experimental worms. Plates were seeded with OP50 *E. coli* as a food source.

### 5.2 Lifespan

Lifespan experiments were conducted at a temperature of 20°C. Survival was measured every day until death. Plates contained 25 μM 5′‐fluorodeoxyuridine (FUdR) to minimize internal hatching of progeny. This concentration of FUdR does not affect wild‐type lifespan (Van Raamsdonk & Hekimi, 2011) and does not affect the lifespan of most, but not all, mutant strains. In the absence of FUdR, all of these strains are still long‐lived and the relative order of the strains is similar (Apfeld & Kenyon, 1999; Feng et al., 2001; Hansen et al., 2007; Hsin & Kenyon, 1999; Kenyon et al., 1993; Lakowski & Hekimi, 1996, 1998; Syntichaki et al., 2007; Van Raamsdonk & Hekimi, 2009; Wong et al., 1995; Yang & Hekimi, 2010). Lifespan experiments were completed with at least three biological replicates and a minimum of 40 worms per strain for each trial. In our experience, this number of worms is sufficient for detecting differences in lifespan that are biologically relevant. Survival data were pooled across multiple trials. Worms with internal hatching or extruded vulva were censored. Raw lifespan data can be found in Table S6.

### 5.3 Stress assays

All stress assays were conducted using at least three biological replicates with a minimum of 20 worms per plate at a temperature of 20°C.

### 5.4 Oxidative stress

For acute oxidative stress, young adult worms were transferred onto freshly poured agar plates containing 300 μM juglone. Survival was measured every 2 h for a total of 10 h. For chronic oxidative stress, young adult worms were transferred onto agar plates containing 4 mM paraquat and 100 μM FUdR. Survival was measured daily until death.

### 5.5 Heat stress

Resistance to heat stress was tested by transferring young adult worms to agar plates and placing plates in 37°C. Survival was measured every 2 h for a total of 10 h.

### 5.6 Osmotic stress

Resistance to heat stress was tested by transferring young adult worms to agar plates containing 450 mM or 500 mM NaCl in the NGM plates. Survival was measured after 48 h.

### 5.7 Anoxic stress

Resistance to anoxic stress was tested by transferring young adult worms to agar plates and putting the plates in BD Bio‐Bag Type A Environmental Chambers (Becton, Dickinson and Company, NJ). Survival was measured after 72 or 96 h.

### 5.8 Bacterial pathogen stress

Resistance to bacterial pathogen stress was performed using the slow‐killing assay as described previously (Campos et al., 2021; Wu et al., 2019). *P. aeruginosa* liquid culture were grown in Luria–Bertani (LB) media overnight and were used to seed NGM plates for the slow‐killing assay. Plates were incubated at 37°C for 24 h and then at 20°C for 24 h. L4 worms were transferred to NGM plates containing 100 mg/L FuDR and grown with OP50 until day 3 of adulthood. Adults were transferred to PA14‐seeded plates containing 20 mg/L FuDR. Assay was conducted at 20°C. Worms were checked daily until death.

### 5.9 RNA sequencing and bioinformatic analysis

RNA sequencing was performed on young adult worms collected from six independent samples for each strain as described previously (Dues et al., 2017). RNA‐seq data are available on NCBI GEO (GSE93724 (Senchuk et al., 2018) GSE110984 (Wu et al., 2018), GSE179825) and were analyzed by the Harvard School of Public Health Bioinformatics core for this paper. For read mapping and expression level estimation, we used an RNA‐seq pipeline from the bcbio‐nextgen project (https://bcbio‐nextgen.readthedocs.org/en/latest/) to process samples. We examined quality of the raw reads using FastQC (http://www.bioinformatics.babraham.ac.uk/projects/fastqc/) and trimmed any reads that contained contaminant sequences and low‐quality sequences with cutadapt (http://code.google.com/p/cutadapt/). We used STAR (Dobin et al., 2013) to align trimmed reads to the Ensembl build WBcel235 (release 90) of the *C. elegans* genome. To check alignment quality, we checked for evenness of coverage, rRNA content, genomic context of alignments, and complexity. For expression quantification, we used Salmon (Patro et al., 2017) for identifying transcript‐level abundance estimates and then used R Bioconductor package tximport (Soneson et al., 2015) for collapsing down to the gene level. To validate sample clustering from the same batches across different mutants, we used principal components analysis (PCA) and hierarchical clustering methods. We used R Bioconductor package DESeq2 (Love et al., 2014) to perform differential gene expression analysis. For each comparison between wild‐type and mutant, a false discovery rate (FDR) threshold of 0.01 was set to identify significant genes. To adjust for batch effects, we included batch as a covariate in the linear model for datasets in experiments that were run across two batches.

### 5.10 Overlapping genes

Lists of the differentially expressed genes from mutant were compared with genes modulated by each stress pathway. Comparisons were made between differentially expressed genes of the same direction of change. The hypergeometric test was used to compute significance of overlap. Venn diagrams were made from the online tool BioVenn (https://www.biovenn.nl/).

### 5.11 Gene ontology analysis

PANTHER Classification System (version 16.0) was used for functional analysis of genes correlated with stress. Statistical overrepresentation analysis of Gene Ontology (GO) terms was performed by inputting the WormBase IDs. Fisher's Exact test was used to calculate false discovery rates to determine the significantly enriched GO terms.

### 5.12 Statistical analysis

Experiments were performed with the experimenter blinded to the genotypes of the worms being tested. A minimum of three biological replicates on different days were performed for each assay. All statistical analyses were performed using GraphPad Prism version 5.01. Survival plots for lifespan assays, oxidative stress assays, and bacterial pathogen stress assays were analyzed using a log‐rank test. Heat stress assays were analyzed using repeated measures ANOVA. Osmotic stress and anoxic stress assays were analyzed using one‐way ANOVA with Dunnett's multiple comparison test. In Figure 1 panels a–e, the results from longitudinal survival assays are presented in bar graph form to facilitate comparison across all of the strains. The full results from these survival assays can be found in Figures S1–S5. Because the supplemental figures include all of the time points and use a different statistical test, a greater number of the mutants exhibit a statistically significant increase in stress resistance in the supplemental figures compared with Figure 1. The conclusions and summary table are based on the results in the supplemental figures as these results take into account all of the time points examined.

## AUTHOR CONTRIBUTIONS

JVR involved in conceptualization and supervision. SKS, AT, ZR, AM, MM, and JVR involved in methodology, investigation, analysis, and visualization. SKS and JVR involved in writing—original draft. SKS, AT, ZR, AM, and JVR involved in writing—review and editing.

## FUNDING INFORMATION

This work was supported by the Canadian Institutes of Health Research (CIHR; http://www.cihr‐irsc.gc.ca/; JVR), and the Natural Sciences and Engineering Research Council of Canada (NSERC; https://www.nserc‐crsng.gc.ca/index_eng.asp; JVR). JVR is the recipient of a Senior Research Scholar career award from the Fonds de Recherche du Québec Santé (FRQS) and Parkinson Quebec. SKS is supported by a FRQS Doctoral Award. ZDR is supported by a FRQS Postdoctoral award. The funders had no role in study design, data collection and analysis, decision to publish, or preparation of the manuscript.

## CONFLICT OF INTEREST

The authors declare that they have no conflict of interest.

## Supporting information


Appendix S1

Table S2

Table S3

Table S4

Table S6

## Data Availability

RNA‐seq data have been deposited on GEO: GSE93724, GSE110984, and GSE179825. All other data and strains generated in the current study are included with the manuscript or available from the corresponding author on request.
