# Supplementary material for: Genetic basis of enhanced stress resistance in long‐lived mutants highlights key role of innate immunity in determining longevity
Source: Aging Cell. 2022 Dec 13;22(2):e13740. doi: 10.1111/acel.13740 (PMC9924947; doi:10.1111/acel.13740)
Supplement: Supplementary file 1 — Appendix S1 [file ACEL-22-e13740-s004.pdf]

Supporting Information for:

Genetic basis of enhanced stress resistance in long-lived mutants highlights key role of innate immunity in determining longevity

Sonja K. Soo[1,2], Annika Traa[1,2], Zenith D. Rudich[1,2], Alibek Moldakozhayev[1,2], Meeta Mistry[3], Jeremy M. Van Raamsdonk[1,2,4,5]

[1]Department of Neurology and Neurosurgery, McGill University, Montreal, Quebec, Canada

[2]Metabolic Disorders and Complications Program, and Brain Repair and Integrative Neuroscience Program, Research Institute of the McGill University Health Centre, Montreal, Quebec, Canada

[3]Bioinformatics Core, Harvard School of Public Health, Harvard Medical School, Boston. MA, USA

[4]Division of Experimental Medicine, Department of Medicine, McGill University, Montreal, Quebec, Canada

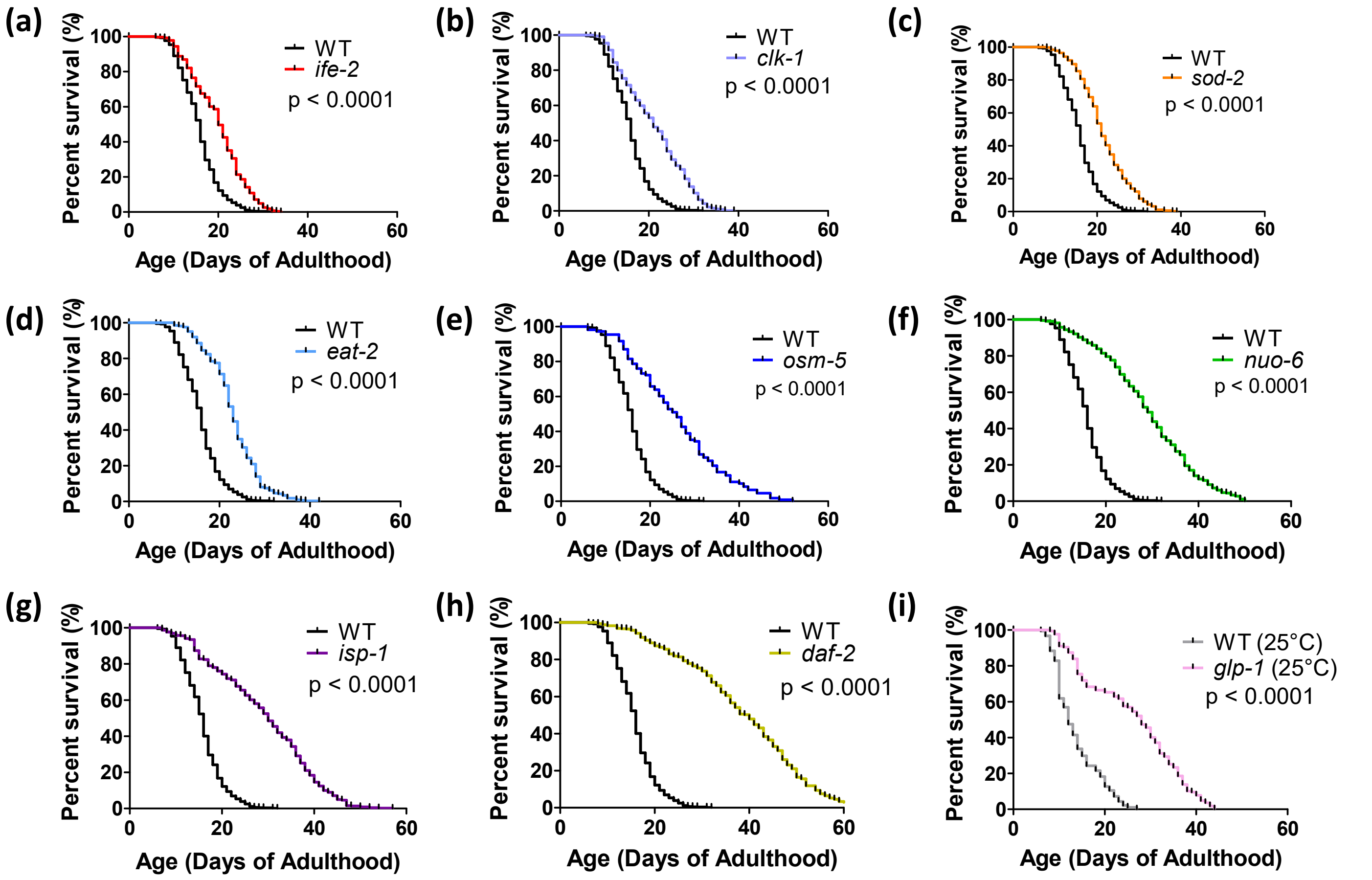

FIGURE S1. Long-lived mutants exhibit different magnitudes of lifespan extension. In order to compare the length of lifespan extension between different long-lived mutants, we measured the lifespan of nine long-lived mutants simultaneously, under the same conditions. As demonstrated previously, *ife-2* (**a**), *clk-1* (**b**) , *sod-2* (**c**), *eat-2* (**d**), *osm-5* (**e**), *nuo-6* (**f**), *isp-1* (**g**), *daf-2* (**h**) and *glp-1* (**i**) mutants all show increased lifespan compared to wild-type control worms. The length of lifespan extension varied from 4 days (25% increase) in *ife-2* worms to 22 days (138% increase) in *daf-2* worms. For *glp-1* worms and their wild-type controls, worms were grown at 25°C during development and were shifted to 20 °C at adulthood. Three biological replicates per strain were performed. Significance was determined using the log-rank test.

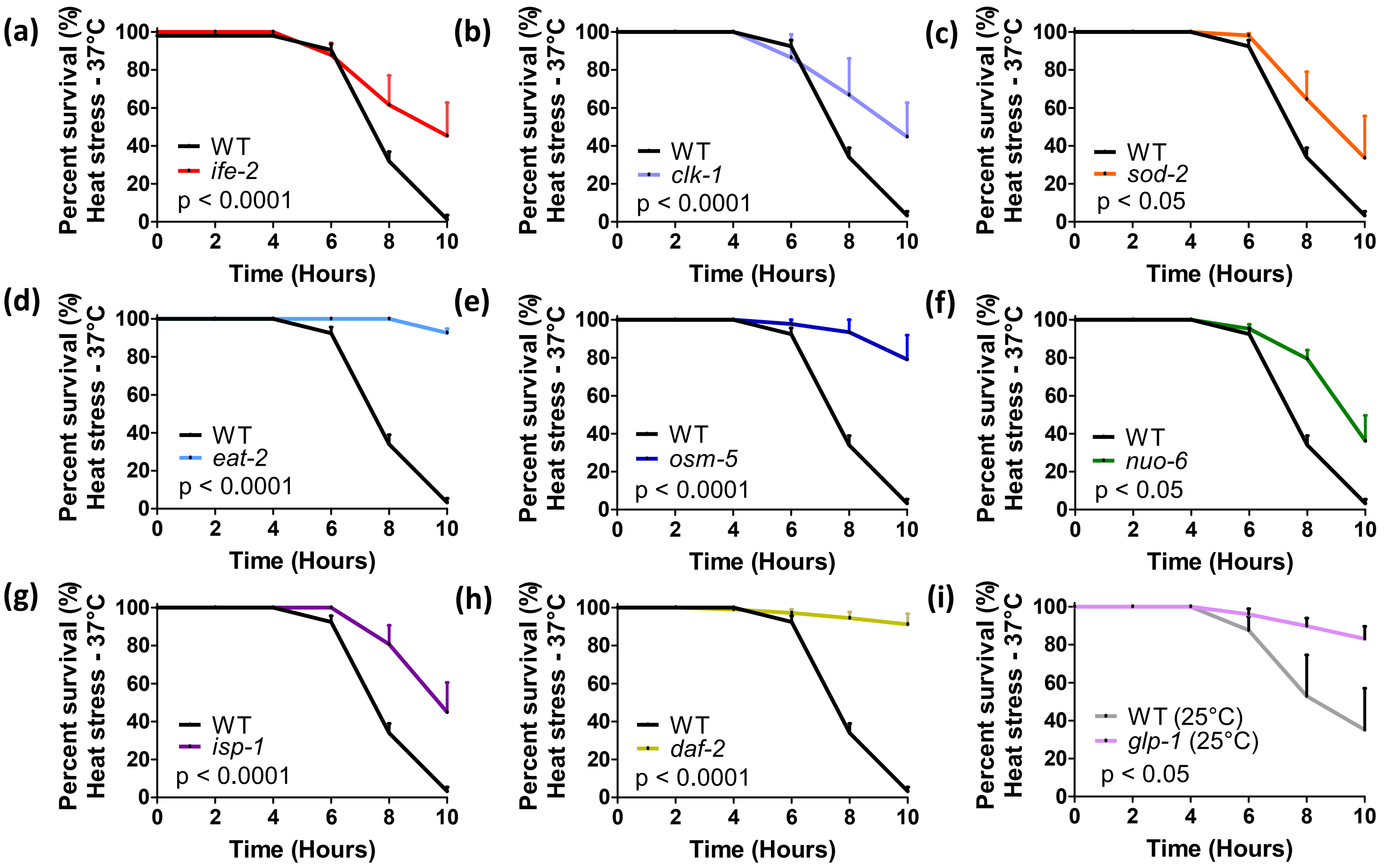

FIGURE S2. All long-lived mutants have increased resistance to heat stress. *ife-2* (**a**), *clk-1* (**b**), *sod-2* (**c**), *eat-2* (**d**), *osm-5* (**e**), *nuo-6* (**f**), *isp-1* (**g**), *daf-2* (**h**) and *glp-1* (**i**) worms show increased resistance to 37°C heat stress compared to wild-type worms. For *glp-1* worms and their wild-type controls, worms were grown at 25°C during development and were shifted to 20 °C at adulthood. Error bars represent SEM. Three biological replicates per strain were performed. Significance was determined using a repeated measures ANOVA.

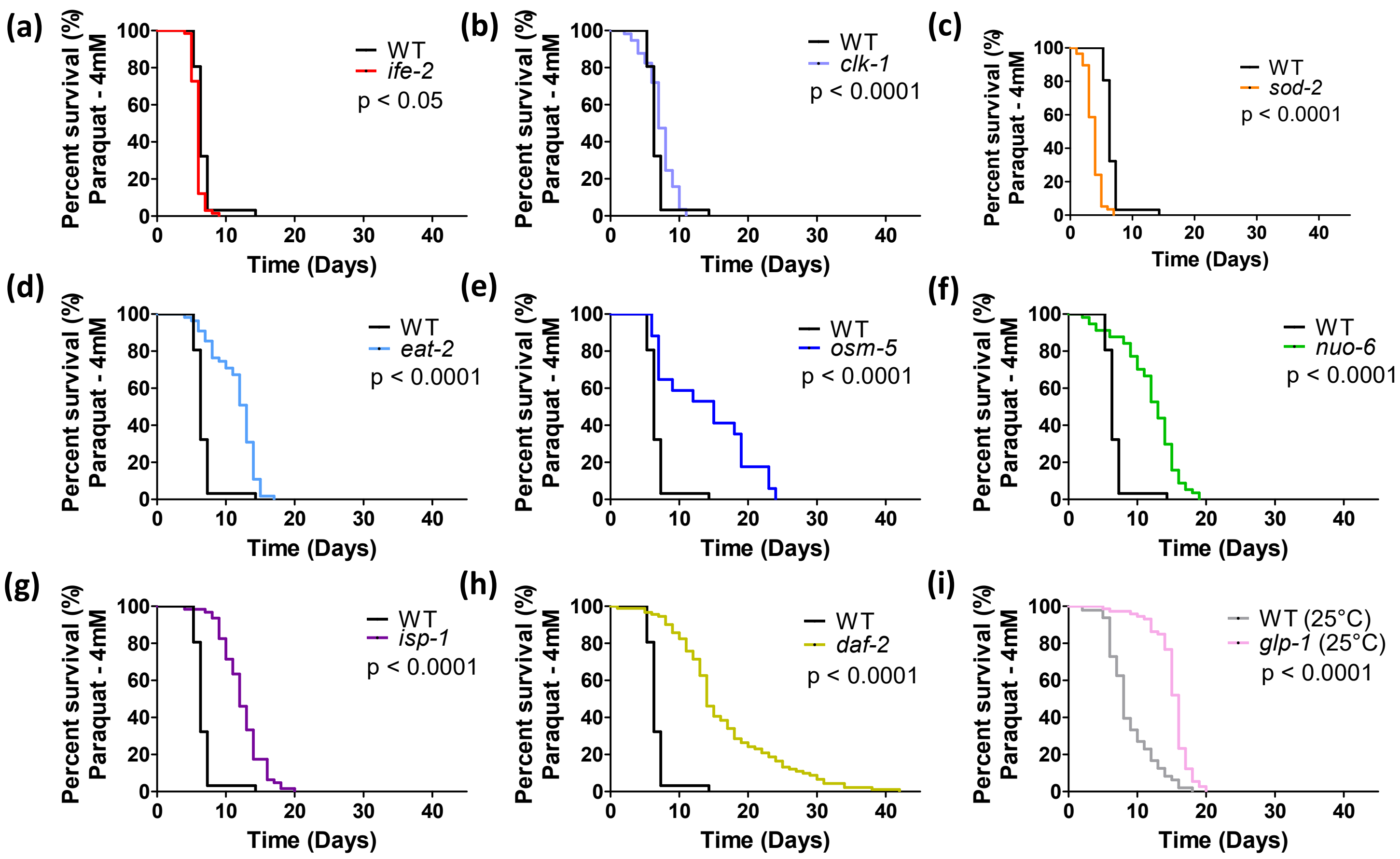

FIGURE S3. Most but not all long-lived mutants exhibit increased resistance to chronic oxidative stress resulting from exposure to paraquat. Resistance to chronic oxidative stress was measured by monitoring survival on plates containing 4 mM paraquat. *ife-2* (**a**) and *sod-2* (**c**) mutants show less resistance to paraquat compared to wild-type worms. *clk-1* (**b**) , *eat-2* (**d**), *osm-5* (**e**), *nuo-6* (**f**), *isp-1* (**g**), *daf-2* (**h**), and *glp-1* (**i**) worms show increased resistance to paraquat compared to wild-type worms. All six of the longest lived strains show increased resistance to paraquat. For *glp-1* mutants and their wild-type controls, worms were grown at 25°C during development and were shifted to 20 °C at adulthood. Three biological replicates per strain were performed. Significance was determined using the log-rank test.

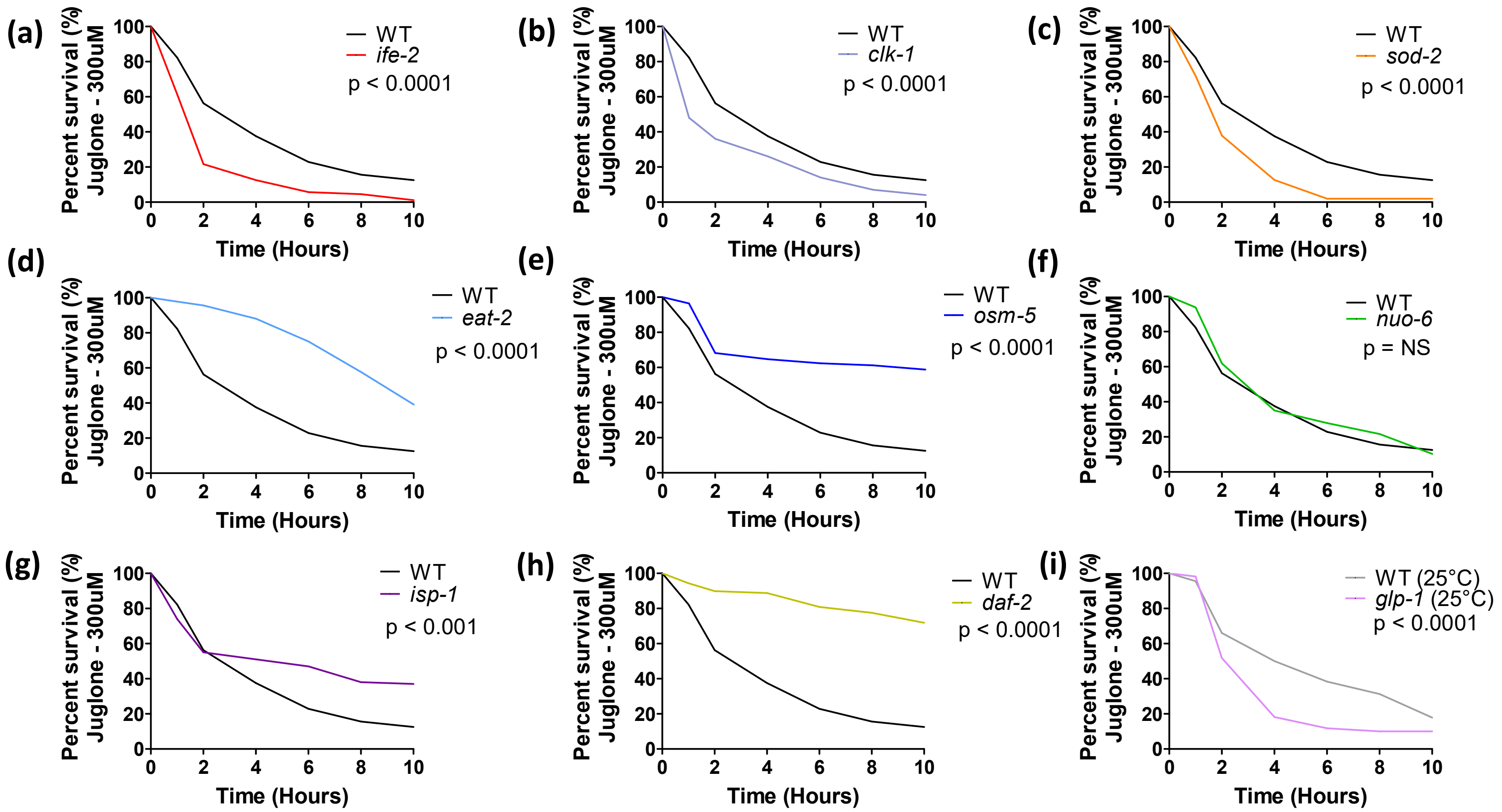

FIGURE S4. Long-lived mutants show variable resistance to acute oxidative stress resulting from exposure to juglone. Resistance to acute oxidative stress was measured by monitoring survival on plates containing 300 μM juglone. *ife-2* (**a**), *clk-1* (**b**), *sod-2* (**c**) and *glp-1* (**i**) mutants show less resistance to juglone compared to wild-type worms. *eat-2* (**d**), *osm-5* (**e**), *isp-1* (**g**), and *daf-2* (**h**) worms show increased resistance to juglone compared to wild-type worms, while *nuo-6* (**f**) worms showed no difference. For *glp-1* mutants and their wild-type controls, worms were grown at 25°C during development and were shifted to 20 °C at adulthood. Three biological replicates per strain were performed. Significance was determined using the log rank test.

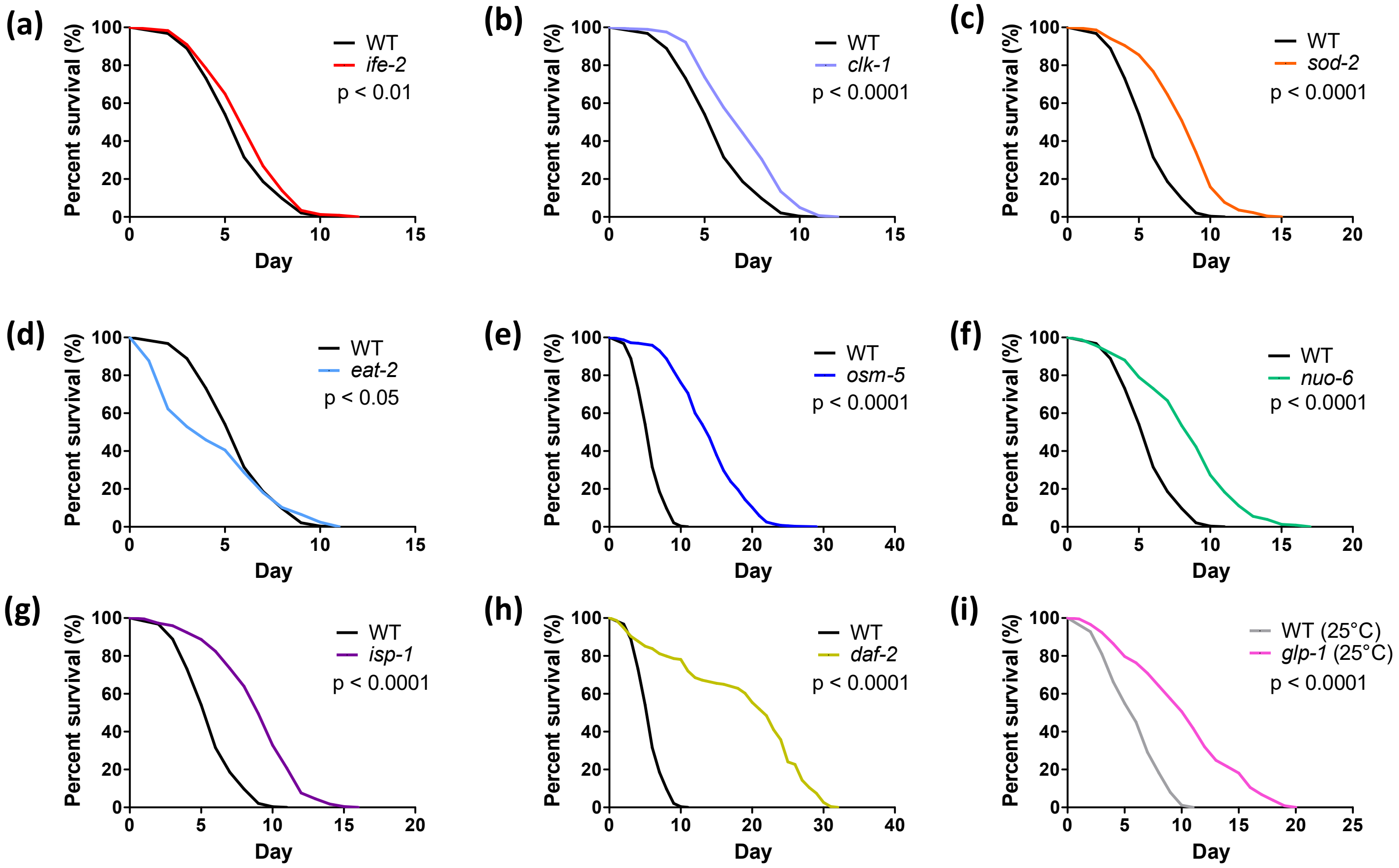

FIGURE S5. Most but not all long-lived mutants exhibit increased resistance to bacterial pathogen stress. Resistance to bacterial pathogen stress was assessed by exposing worms to *Pseudomonas aeruginosa* strain PA14 in a slow kill assay. While *eat-2* (**d**) worms show less resistance to PA14 compared to wild-type worms, *ife-2* (**a**), *clk-1* (**b**), *sod-2* (**c**), *osm-5* (**e**), *nuo-6* (**f**), *isp-1* (**g**), *daf-2* (**h**) and *glp-1* (**i**) mutants all show increased resistance to PA14 compared to wild-type worms. For *glp-1* worms and their wild-type controls, worms were grown at 25°C during development and were shifted to 20 °C at adulthood. Three biological replicates per strain were performed. Significance was determined using the log-rank test.

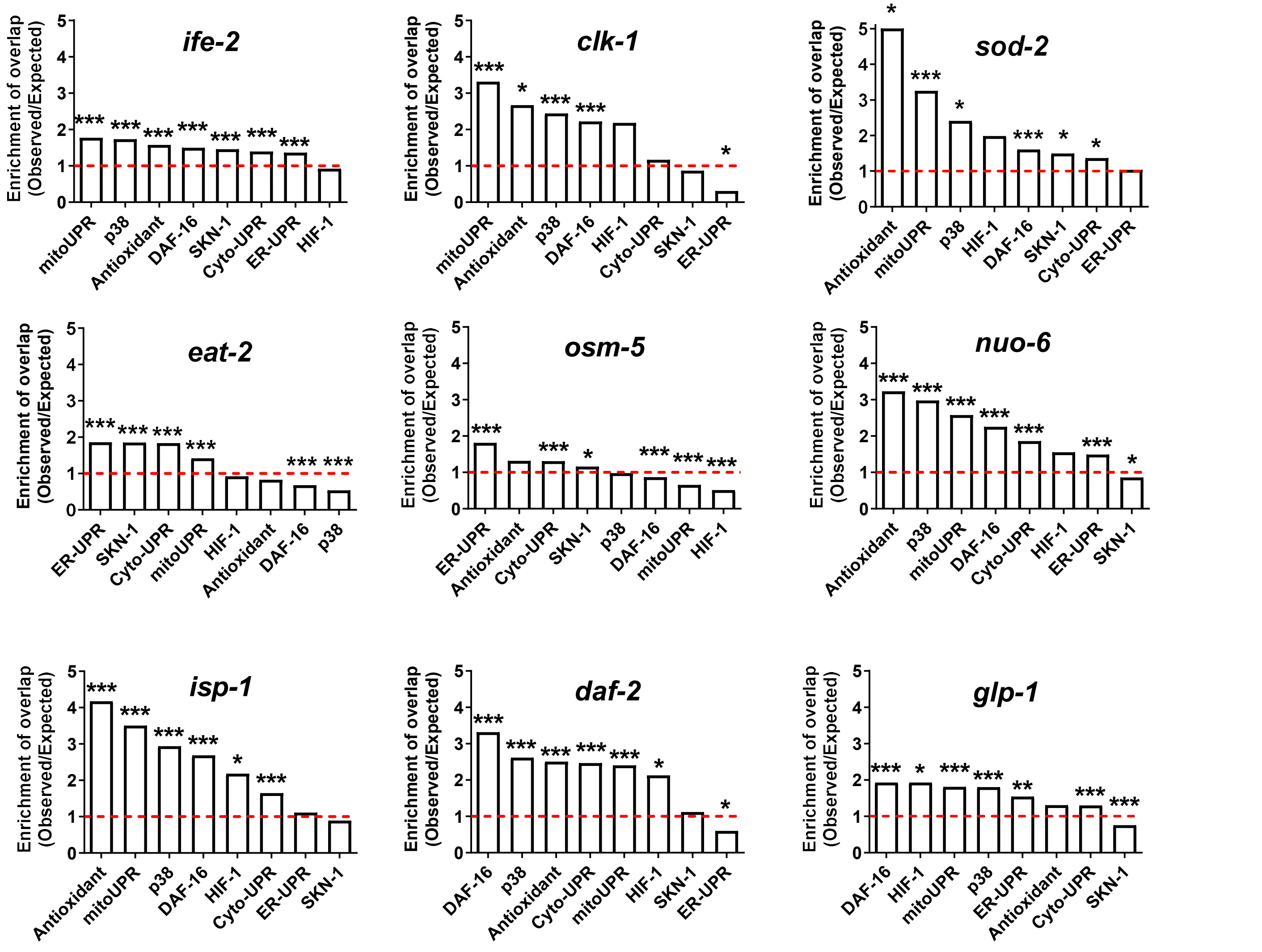

FIGURE S6. Relative contribution of different stress response pathways in long-lived mutants. Gene expression in the long-lived mutant strains was examined by RNA sequencing (RNA-seq) of six biological replicate per genotype of pre-fertile day 1 young adult worms. Differentially expressed genes that are significantly upregulated in the long-lived mutant strains were compared to genes that are upregulated by activation of different stress response pathways including the DAF-16-mediated stress response (DAF-16), the p38-regulated innate immune signaling pathway (p38), the HIF-1-mediated hypoxia response (HIF-1), the SKN-1-mediated oxidative stress response (SKN-1), the mitochondrial unfolded protein response (mitoUPR), the cytoplasmic unfolded protein response (Cyto-UPR), the ER unfolded protein response (ER-UPR), and antioxidant gene expression (Antioxidant). For each stress pathway, the ratio of the observed number of overlapping genes with the long-lived mutant to the expected number of overlapping genes if picked randomly was determined. All of the long-lived mutants showed a significant enrichment of genes involved in at least three stress response pathways. For each mutant, the stress response pathways are arranged in descending order of observed overlapping genes/expected overlapping genes.

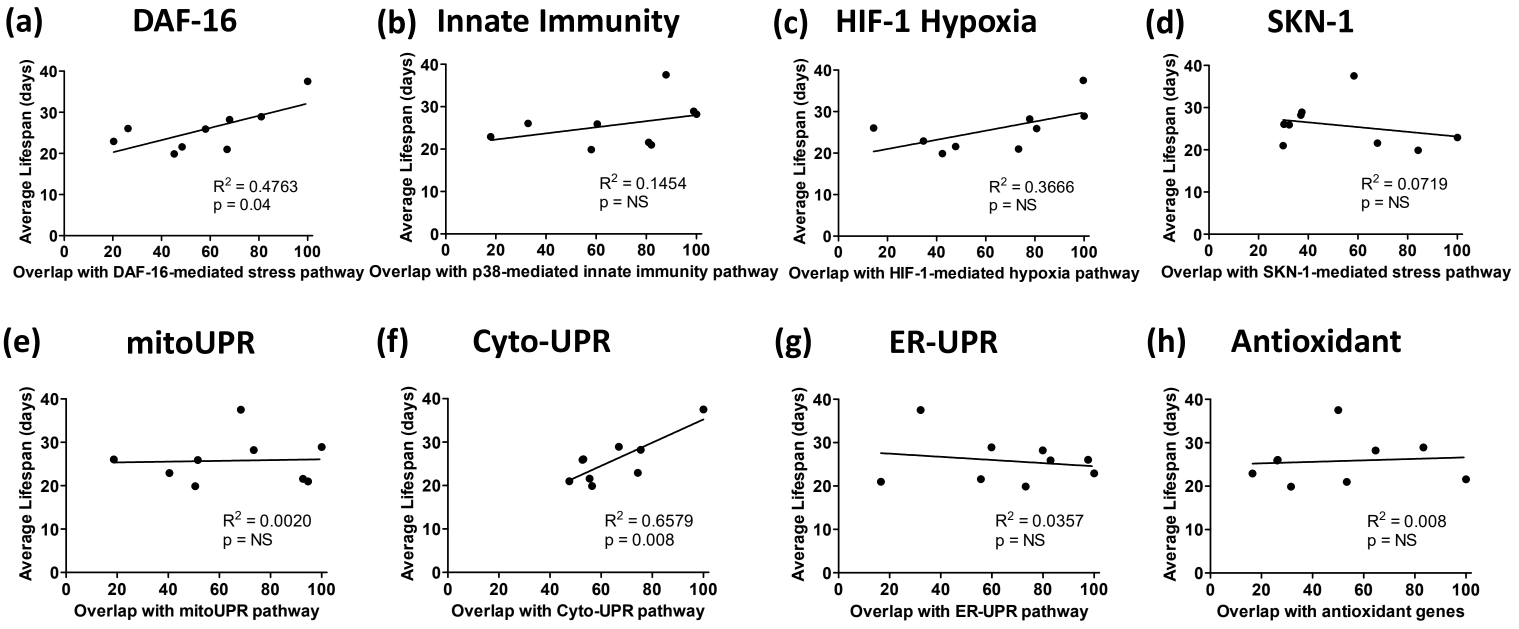

FIGURE S7. Degree of overlap with genes in the cytoplasmic unfolded protein response pathway and DAF-16-mediated stress response pathway are correlated with lifespan. While there is a significant degree of overlap between genes involved in stress response pathways and genes upregulated in long-lived mutants, there is only a significant correlation between the degree of overlap and the magnitude of lifespan extension for the DAF-16-mediated stress response pathway and the cytoplasmic unfolded protein response.

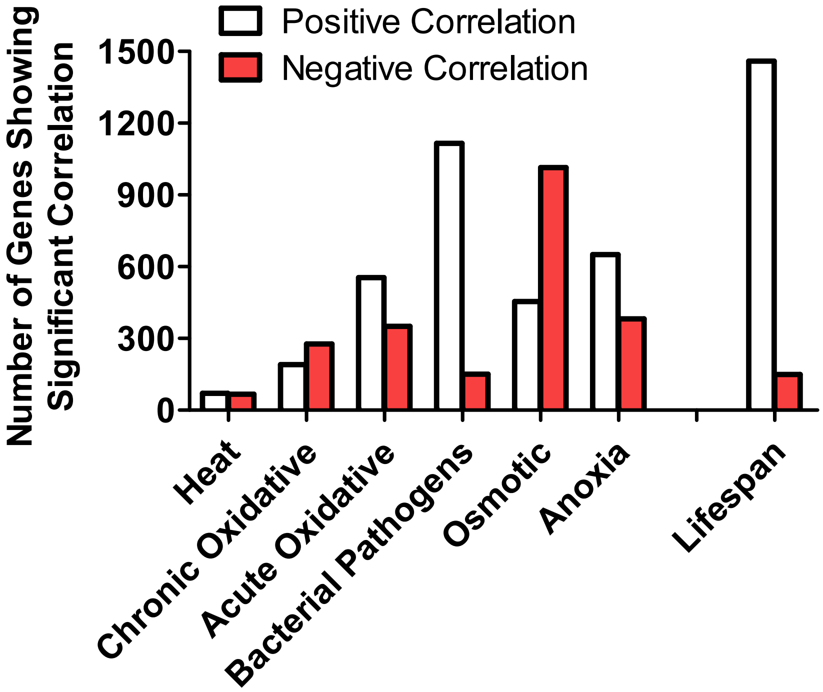

FIGURE S8. Genes correlated with resistance to different external stressors. The expression levels of all of the genes in the genome were determined by RNA sequencing in nine long-lived mutants. These expression levels were compared to the survival of these strains when exposed to different types of stress to determine which genes are correlated with stress resistance. There are numerous genes that exhibit either a positive or negative correlation with each type of stress resistance indicating a strong influence of genetics on stress resistance. Complete lists of genes that are significantly correlated with each type of stress resistance can be found in **Table S3** .

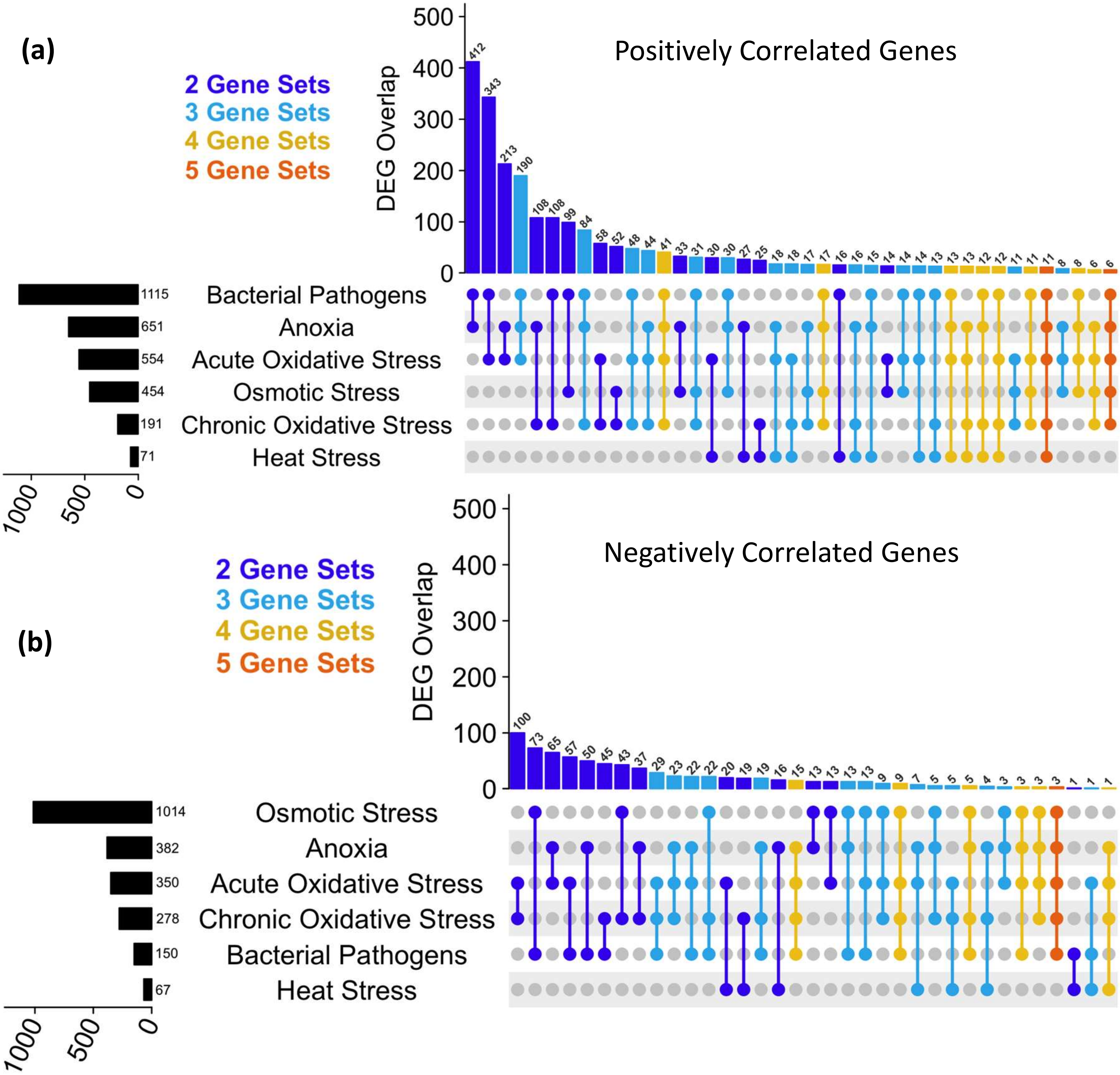

FIGURE S9. Genes correlated with resistance to different external stressors. To determine the extent to which genes correlated with stress would be correlated with multiple types of stress resistance or be specific to certain types of stress resistance and to determine which types of stress resistance show the greatest overlap of correlated genes, we compared genes that showed a significant positive or negative correlation to different types of stress. (**a**) An inclusive UpSetR plot comparing genes positively correlated with each type of stress resistance shows that there are many genes that are positively correlated with 2 or more types of stress resistance. (**b**) An inclusive UpSetR plot comparing genes negatively correlated with each type of stress resistance shows that there are also multiple genes that are negatively correlated with 2 or more types of stress resistance. For panels A and B, the gene sets for each individual stressor are listed on the left and ordered by size from top to bottom. The height of each bar and the number above the bar indicate the number of genes in common between the gene sets indicated by the dots below the plot. The complete lists of genes that are significantly correlated with each type of stress resistance can be found in **Table S3** .

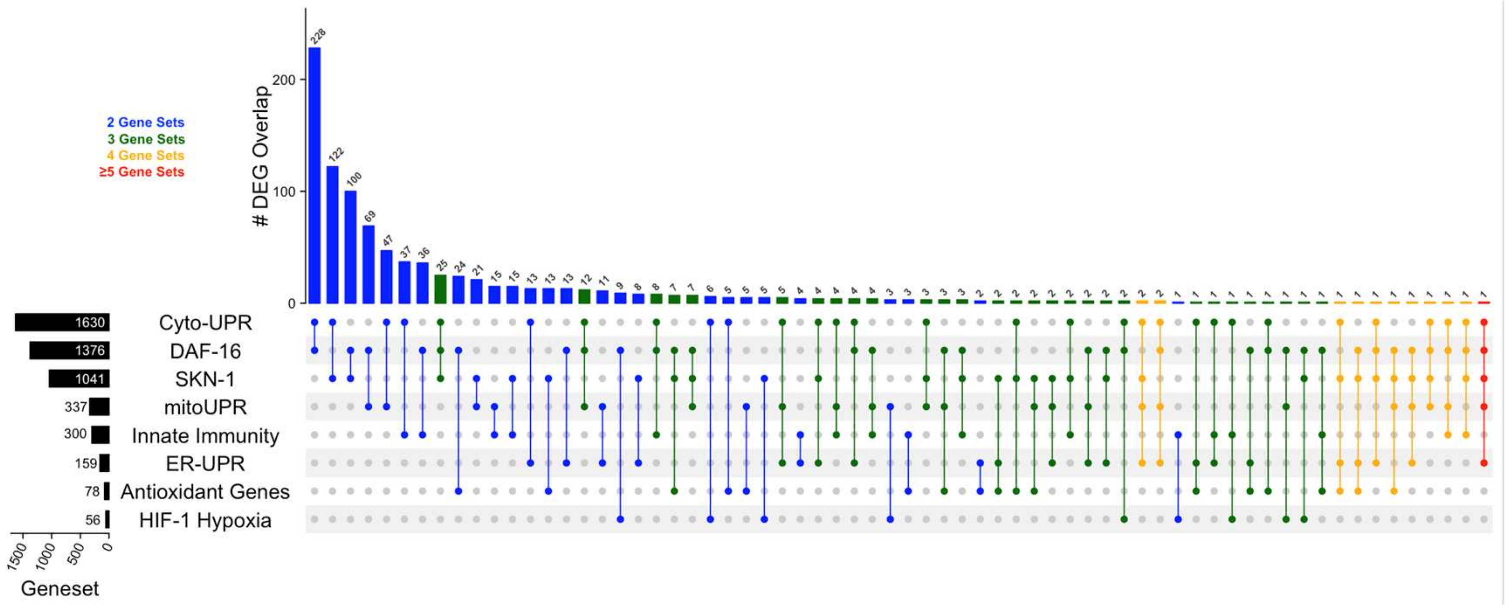

FIGURE S10. Genes upregulated by activation of stress response pathways exhibit partial overlap with other stress response pathways. This inclusive UpSetR plot compares genes that are upregulated by activation of each stress response pathway. Stress response pathways are listed on the left in order of number of upregulated genes from top to bottom. The number of genes that are upregulated by activation of each pathway is indicated by black bars and associated numbers. On the main plot, the height of each bar and the number above the bar indicate the number of genes in common between the gene sets indicated by the dots below the plot. The complete lists of genes that are upregulated by activation of each stress response pathway can be found in **Table S2**. Cyto-UPR = cytoplasmic unfolded protein response; DAF-16 = DAF-16-mediated stress response pathway; SKN-1= SKN-1-mediated oxidative stress response pathway; mitoUPR = mitochondrial unfolded protein response; Innate immunity = p38-mediated innate immune pathway; ER-UPR = endoplasmic reticulum unfolded protein response; HIF-1 Hypoxia = HIF-1-mediated hypoxia pathway.

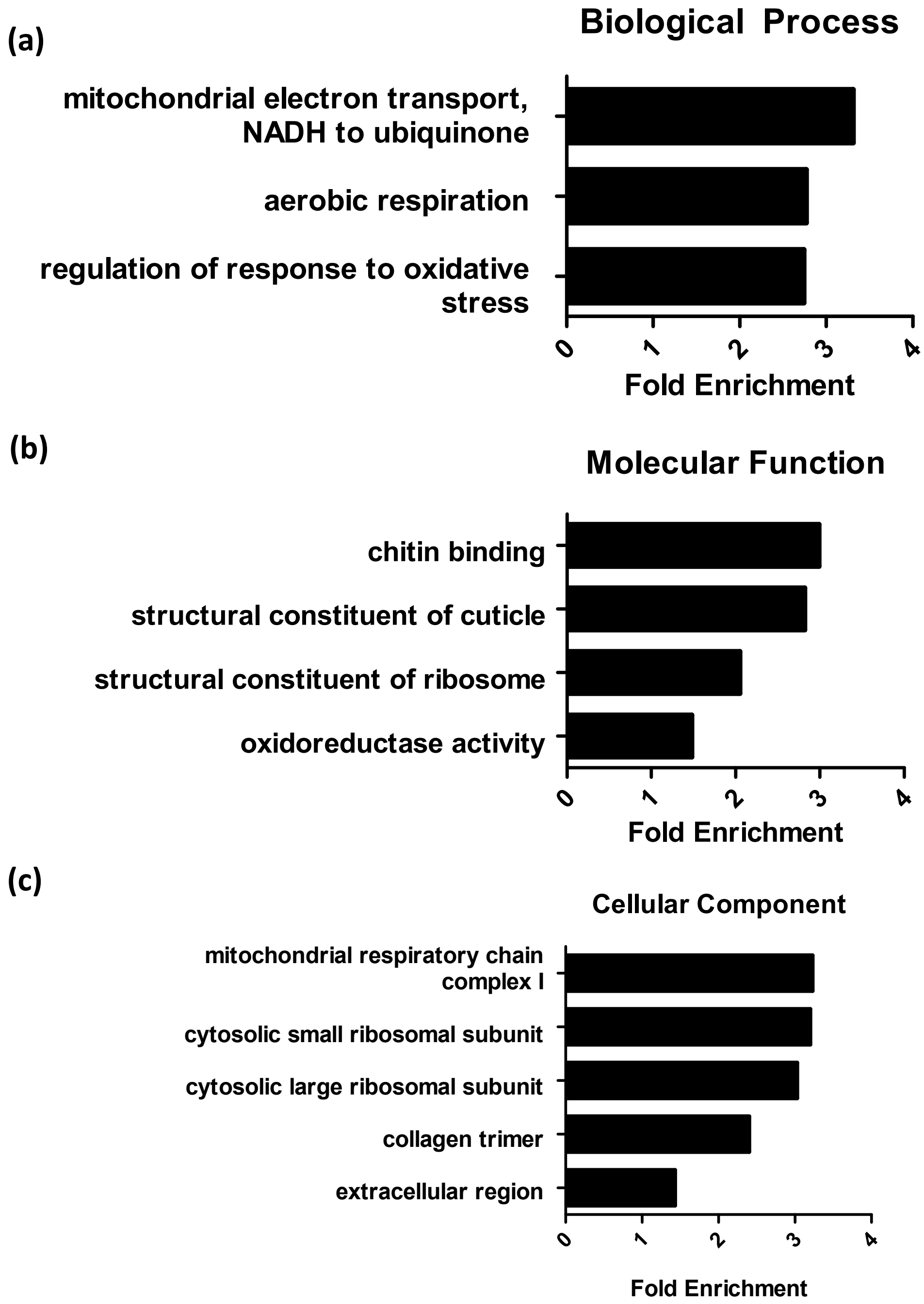

FIGURE S11. Enrichment analysis of genes correlated with resistance to stress reveals a role for mitochondrial function and structures providing a barrier to the environment in stress resistance. To determine the functional classes that are overrepresented in genes correlated with at least one type of stress, we used the statistical overrepresentation test of Gene Ontology (GO) terms with PANTHER Classification System (version 16.0) and compared the genes to the *C. elegans* genome. PANTHER recognized 2997 genes and found 3 significantly enriched classes for Biological Process (**a**), 4 significantly enriched classes for Molecular Function (**b**), and 5 significantly enriched classes for Cellular Component (**c**).

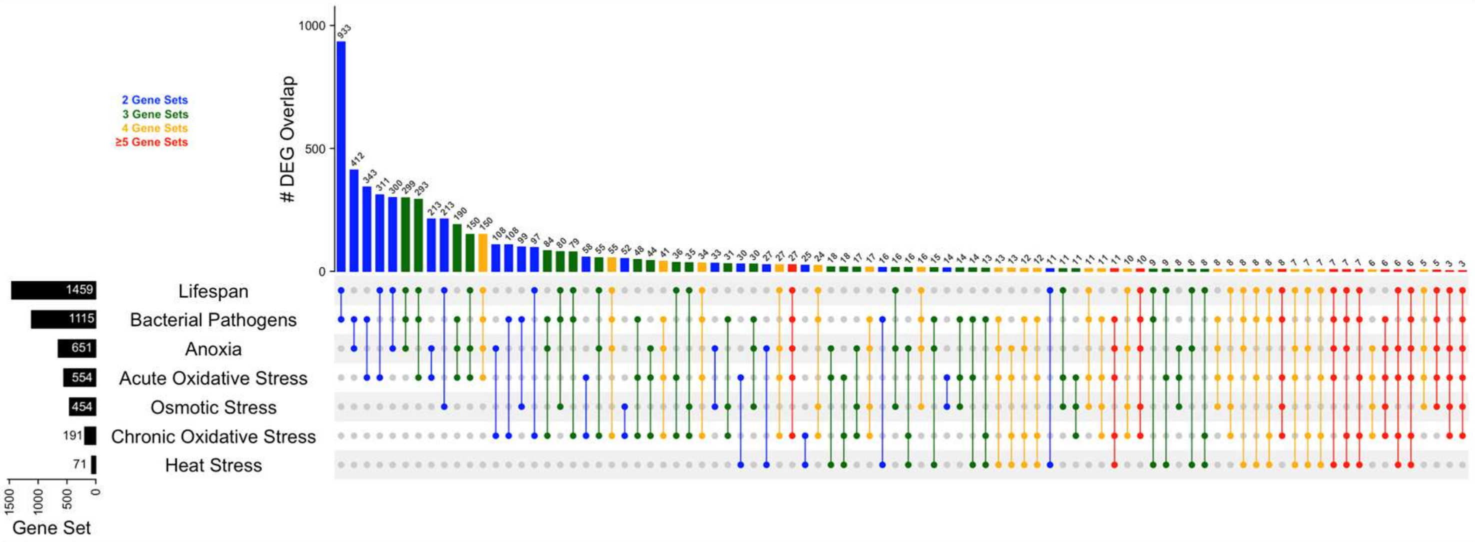

Figure S12. Highly significant overlap between genes the are correlated with resistance to stress and genes that are correlated with lifespan extension. Inclusive UpSetR plot comparing genes correlated with lifespan and genes correlated with different types of stress resistance including bacterial pathogens (*P. aeruginosa*), anoxic stress (72-96 hours), acute oxidative stress (300 µM juglone), chronic oxidative stress (4 mM paraquat), osmotic stress (450-500 mM NaCl), and heat stress (37°C). Black bars on the left indicate the number of genes in each gene set. Full lists of genes correlated with each type of stress resistance can be found in **Table S3**. Bars indicate the number of genes in common between the gene sets indicated by the dots below the plot. The exact number of genes in each intersection is shown above the graph. Intersections involving two gene sets are shown in blue. Intersections involving 3, 4 or 5-6 gene sets are shown in green, yellow or red, respectively.

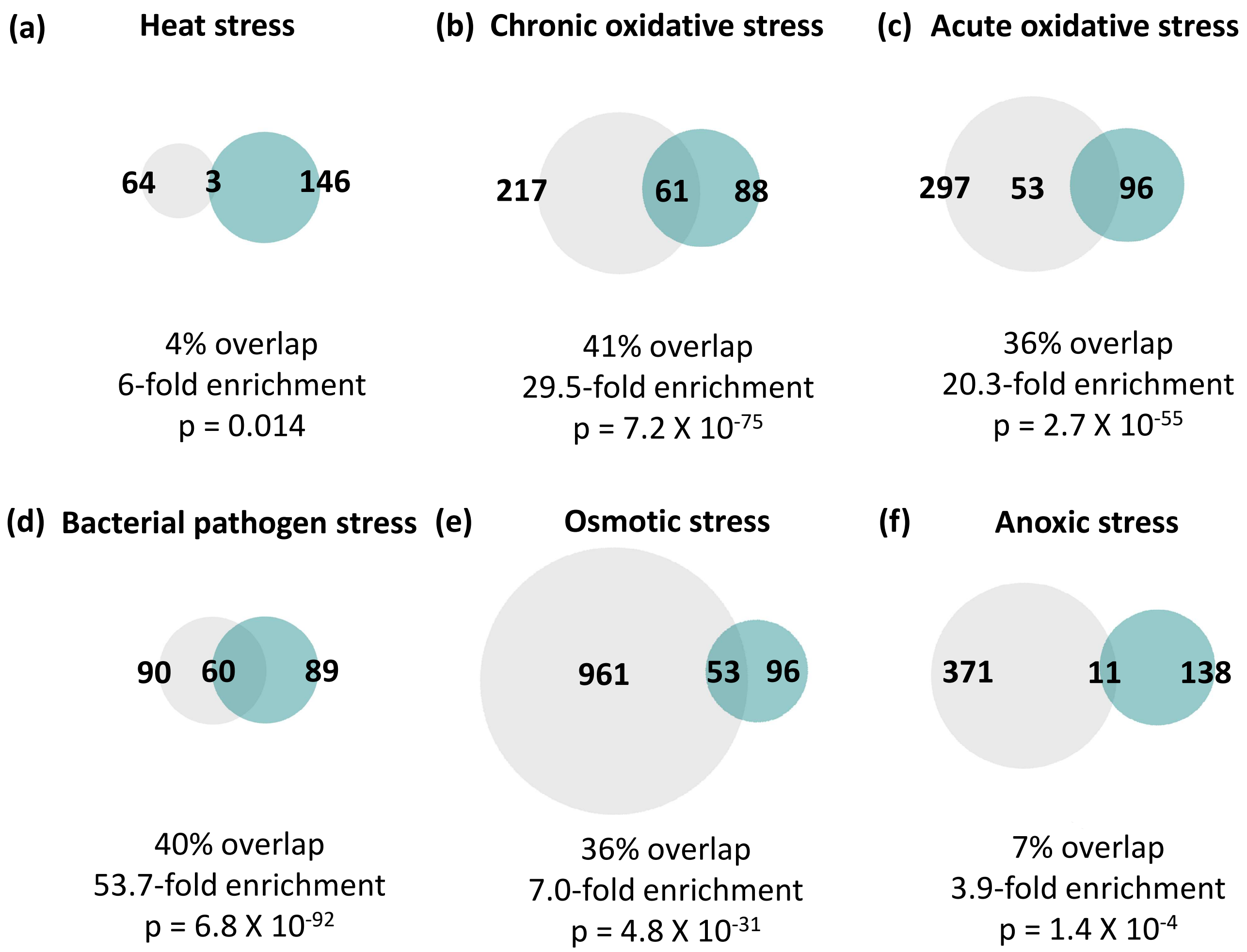

FIGURE S13. Highly significant overlap between genes the are negatively correlated with resistance to stress and genes that are negatively correlated with lifespan extension. In examining the degree of overlap between genes that have a significant negative correlation with lifespan and genes that exhibit a significant negative correlation with resistance to stress, we observed a significant degree of overlap with each of the six types of stress resistance that we examined including heat stress (**a**; 37°C), chronic oxidative stress (**b**; 4 mM paraquat), acute oxidative stress (**c**; 300 µM juglone), bacterial pathogen stress (**d**; *P. aeruginosa*), osmotic stress (**e**; 450-500 mM NaCl), and anoxia stress (**f**; 72-96 hours). The degree of enrichment ranged from 3.9 fold up to 53.7 fold with percent overlap between 4% and 41%. The most highly significant overlap with genes negatively correlated with lifespan was with genes negatively correlated with bacterial pathogen stress survival.

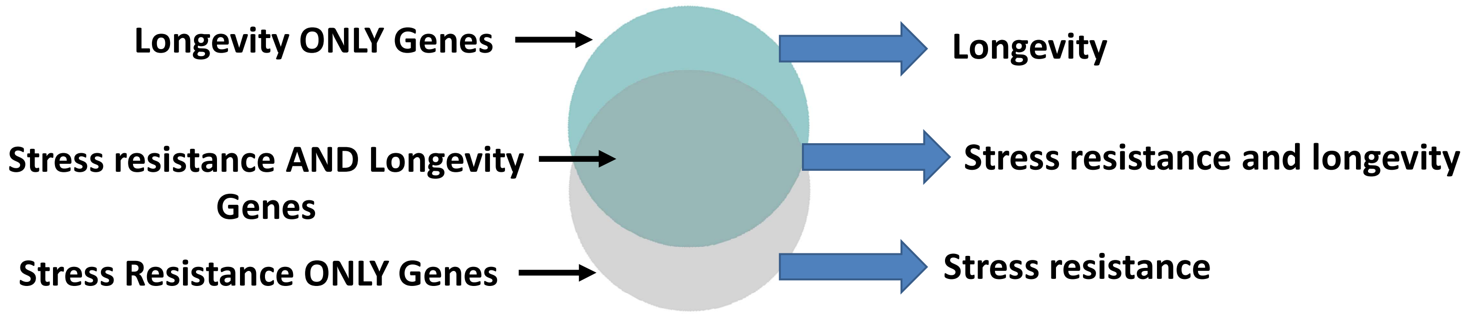

FIGURE S14. Model for relationship between stress resistance and lifespan. The genetic factors contributing to stress resistance and lifespan share a high degree of overlap, but there are also groups of genes that contribute only to one phenotype or the other. Modulating genes that contribute to both stress resistance and longevity will affect both phenotypes and account for the significant correlation that is observed between these two phenotypes. Modulating "Longevity ONLY genes" or "Stress resistance ONLY genes" affects longevity or stress resistance independently of the other, thereby allowing for these phenotypes to be experimentally dissociated.

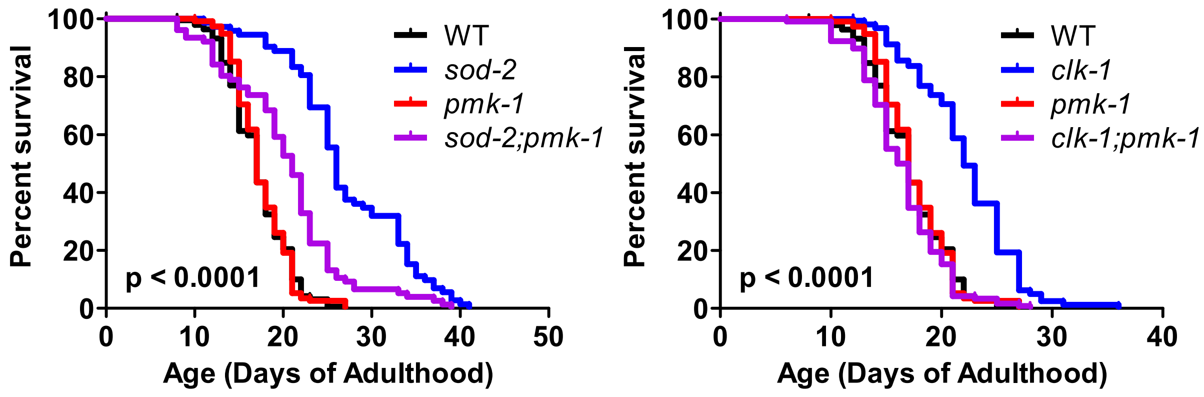

FIGURE S15. PMK-1/p38 is required for the long lifespan of *sod-2* and *clk-1* mutants. To assess the role of the p38-mediated innate immune signaling pathway in the extended longevity of *sod-2* and *clk-1* mutants, we disrupted *pmk-1* using a genetic deletion and examined the effect on lifespan. While *pmk-1* deletion had no effect on wild-type lifespan, it resulted in a significant decrease in *sod-2* and *clk-1* lifespan. This indicates that the innate immune signaling pathway is specifically required for the extended longevity of *sod-2* and *clk-1* mutants. Statistical significance was assessed using the log-rank test.

Table S1. Summary of stress resistance in long-lived mutant strains. "=" indicates no significant difference from wild-type "-" indicates decreased compared to wild-type; "+" indicates increased compared to wild-type; "++" indicates markedly increased compared to wild-type. Markedly increased was defined empirically for each stress as follows: heat stress – greater than 60% survival at 10 hours; chronic oxidative stress – average survival of 13 days or more; acute oxidative stress – greater than 40% survival at 10 hours; bacterial pathogen stress – average survival of 12 days or more; osmotic stress – greater than 60% survival on 500 mM NaCl; anoxic stress – greater than 30% survival at 96 hours.

Strain	Lifespan Extension	Acute oxidative Stress	Chronic oxidative Stress	Heat Stress	Anoxic Stress	Osmotic Stress	PA14 Stress
ife-2	26.3%	-	-	+	=	-	=
clk-1	33.4%	-	+	+	=	=	+
sod-2	37.2%	-	-	+	=	=	+
eat-2	45.6%	+	+	++	=	--	-
osm-5	65.4%	++	++	++	++	++	++
nuo-6	79.2%	=	+	+	-	++	+
isp-1	83.8%	+	+	+	=	++	+
glp-1	89.2%	-	++	++	++	++	+
daf-2	138.4%	++	++	++	++	++	++

Table S5. Effect of RNAi knockdown of genes correlated with stress resistance on resistance to external stressors. ↓ = decreased survival, ↑ = increased survival, NS = effect on survival was not significant. Stress correlate indicates external stressors for which the expression level of the gene exhibited a significant, positive correlation.

Gene	Stress Correlate	Heat Stress	Chronic Oxidative Stress	Acute Oxidative Stress	Bacterial Pathogen Stress	Osmotic Stress	Anoxic Stress
daf-16	Positive Control	↓	↓	↓	↓	↓	↓
F40D4.11	ALL	↑	NS	NS	NS	↓	NS
R08D7.7	ALL	↑	NS	NS	NS	NS	NS
Y75B8A.33	ALL	NS	NS	NS	↓	↓	NS
C30G12.1	ALL	NS	NS	↓	↓	↓	NS
W04G5.8	PQ	↓	NS	↓	NS	NS	NS
F42A6.5	Heat, Juglone	NS	NS	NS	NS	NS	NS
F55D12.5	PQ	↑	NS	NS	NS	↓	NS
K08H10.4	Juglone, PA14, Anoxia	↓	NS	NS	NS	↓	NS
C13B7.3	PA14, Juglone	↑	↓	↓	NS	NS	NS
T01G9.2	Osmotic	NS	NS	NS	NS	↓	NS
F12A10.7	Anoxic, PA14	NS	NS	NS	↓	NS	NS
